# A Review of the Pharmacological Potential of *Spatholobus suberectus* Dunn on Cancer

**DOI:** 10.3390/cells11182885

**Published:** 2022-09-15

**Authors:** Feng Zhang, Kumar Ganesan, Qingqing Liu, Jianping Chen

**Affiliations:** 1 School of Chinese Medicine, LKS Faculty of Medicine, The University of Hong Kong, Hong Kong, China; 2 Shenzhen Institute of Research and Innovation, The University of Hong Kong, Shenzhen 518057, China

**Keywords:** *Spatholobus suberectus* Dunn, phytochemistry, pharmacological activity, mechanism, cancer prevention

## Abstract

*Spatholobus suberectus* Dunn (SSD) has been extensively employed in Traditional Chinese Medicine to treat several ailments. SSD and its active compounds are effective therapeutic agents for treating a variety of diseases with negligible side effects. Therefore, we aimed to investigate its phytochemistry, pharmacology, and potential therapeutic effects exclusively in cancer prevention and treatment. Phytochemical and pharmacological information was collected and arranged in a rational order. SSD has been frequently attributed to having antioxidant, anti-diabetic, anti-inflammatory, hematopoietic, neuroprotective, antimicrobial, and anticancer properties. Evidence has indicated that the bioactive constituents in SSD have attracted increasing scientific attention due to their preventive role in cancers. Further, the present review provides the current information on the health implications of SSD, thus allowing for future clinical trials to explore its restorative benefits. All data of in vitro and animal investigations of SSD, as well as its effect on human health, were obtained from an electronic search and library database. The diverse pharmacological potential of SSD provides an opportunity for preclinical drug discovery, and this comprehensive review strongly indicates that SSD is an excellent anti-tumorigenic agent that modulates or prevents breast cancer.

## 1. Introduction

Medicinal plants are a complementary source for humans that provide better health to humans and the environment. Since prehistoric times, herbal products have been used worldwide as the principal source of curative drugs [1,2,3]. They are the source of outstanding and effective bioactive compounds, which may be used as medicines [4,5,6]. According to recent facts, the plant kingdom comprises approximately 250,000 plant species, of which only about 15% have been investigated for the treatment of several illnesses [7,8,9]. To understand their pharmacological role in disease prevention, the study of the phytochemical properties of diverse plants and the analogs of their bioactive compounds is of critical importance.

**Figure 1:**
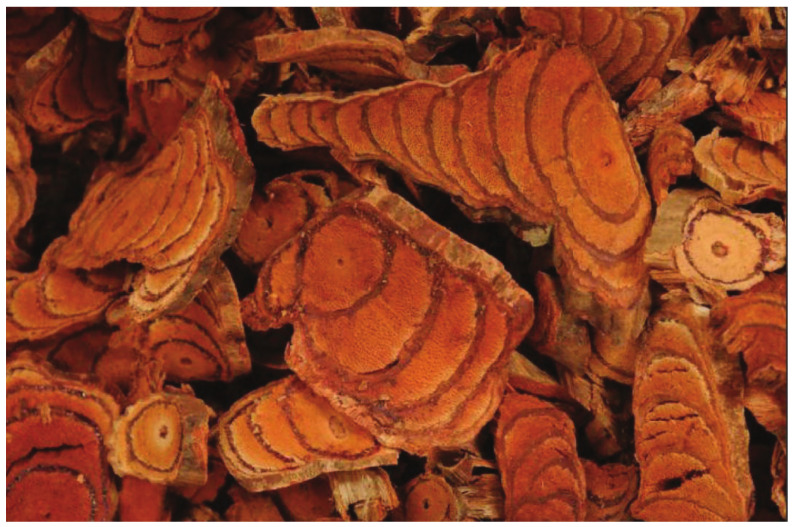
The dry stem of SSD.

*Spatholobus suberectus* Dunn (SSD, Leguminosae) is a perennial woody vine, indigenous to tropical and subtropical forests in China and other Southeast Asian countries [10]. The vine stem of SSD is called “Jixueteng” (literally means ‘chicken blood vines’) in Chinese, due to the blood-like outflow from its vine stem when it is injured (Figure 1). The vine stem of SSD has been widely employed in Chinese Medicine to treat hematopoiesis, anemia, rheumatism, and menoxenia in Chinese society for nearly 1000 years [11]. It is most commonly used as a food additive in southern Asian regions for wine, tea, and soup. In clinical practice, herbal medicine is highly effective in terms of its bioavailability and dose effectiveness, as it contains active ingredients with reliable bioavailability. The crude extract from SSD is thus used in a number of patented Chinese medicines, and the demand for the crude resource is increasing rapidly today. However, unlike other legume families, the seedling growth proportion of SSD is relatively weak; the fruit drops off too early, resulting in poor reproductive ability [12]. SSD usually takes 7 years to develop and yield effective drugs, which can then be used as medicines. Over the past decades, SSD has been recognized as an endangered plant species and has been documented on the Red List of Biodiversity in China, as a consequence of its low growth rate and man-made activities, including deforestation and overexploitation [10]. Furthermore, the market demand for SSD is massive, and the wild resources can no longer meet these mounting requirements. Thus, the Ministry of Ecology and Environment encourages the artificial planting of SSD in highland areas of Xishuangbanna, southwest Yunnan, China. This area is characterized by a distinct climate, sporadic rainy events, and dry seasons, which can be used in order to cultivate surplus numbers of SSD.

SSD contains several bioactive substances that have gained increased scientific interest recently. These bioactive substances are efficient therapeutic agents used to treat a range of illnesses. Hence, in this review, we aimed to focus on their phytochemistry, pharmacology, and potential therapeutic effects in cancer prevention with their molecular mechanisms. All data on phytochemistry, pharmacology, and potential therapeutic effects in cancer prevention were collected from electronic searches of library databases. An electronic search was conducted using PubMed, Science Direct, and Google Scholar by searching for the keywords SSD with “phytochemicals” AND “bioactive compounds” AND “flavonoids” AND “polyphenols” OR “cancers” OR “biological activity” in “Title/Abstract/Keywords” with the date from January 2011 to August 2022, to identify all published studies (in vitro, in vivo, and clinical) that have investigated the connection between SSD and its various beneficial effects. 

## 2. Phytochemistry 

SSD comprises distinguished polyphenol compounds, including flavonoids, carotenoids, chalcone, coumestans, lignans, and phenolic acid [13]. The phenolic contents of SSD are relatively higher than that of many other medicinal plants [14,15,16]. Several analytical methods have been used to isolate, purify, and characterize the chemical compounds from SSD [17,18,19,20]. Several bioactive chemicals in SSD can be identified by HPLC chromatographic fingerprinting. The polyamide column chromatography method is also used to separate polyphenols in SSD. This is followed by the HPLC method to determine the flavonoid components, which enables quality control assurance in identifying the compounds in SSD [21]. Ultraviolet spectrophotometry applications in vanillin–acid assays and the aluminum nitrate colorimetric method are also employed to determine the content of condensed tannins and total flavonoids in SSD [22,23,24,25]. 

Flavonoids, particularly flavanols, are recognized as the most abundant substances in SSD [26]. SSD is rich in catechin and its analogs in the descending order of epicatechin, gallocatechin, and catechin [26]. After a series of extractions, a subfraction of vine stem extracts yield formononetin (39.418%, *w*/*w*), daidzein (9.504%, *w*/*w*), genistein (7.204%, *w*/*w*), calycosin (4.555%, *w*/*w*), glycyrrhizin (4.369%, *w*/*w*), prunetin (3.288%, *w*/*w*), epicatechin (0.927%, *w*/*w*), *p*-hydroxy benzoic acid (0.486%, *w*/*w*), and protocatechuic acid (0.704%, *w*/*w*) [27,28]. Our lab has isolated five isoliquiritigenin analogs with a novel methylene-bridged bischalcone, 3′,4′,5′,4″-tetramethoxychalcone, from SSD, which have significant cytotoxicity against human BC cells [29].

About 57 chemical ingredients in the SSD stem were isolated by the effective method of combining Ultra-Fast Liquid Chromatography and Tandem Mass Spectrometry. The content of chemicals detected in the descending order was isoflavones, flavanols, phenolic acid esters, terpenoids, lignans, and coumarins. Among a pool of chemicals, (-)-gallocatechin, (-)-catechin, (-)-epicatechin, biochanin A, (-)-epiafzelechin, 4,7,2′-trihydroxy-4′-methoxyisoflavanol, β-sitosterol, dihydrocajanin, and maackiain exhibited the highest concentrations in crude extracts [30]. Different analytical techniques can be used to determine the content of bioactive compounds that distinguish the quality and quantity of SSD. A total of 16 compounds were found in SSD as identified by HPLC-MS/MS [31]. Four active flavonoids, viz., protocatechuic acid, catechin, gallocatechin, and formononetin, were determined in the plasma of rats by a UPLC-MS/MSn method, and these active principles were successfully employed in a pharmacokinetics investigation employed after the oral administration of SSD [32]. Diversified compounds from SSD have been identified and classified based on the class, subclass, and structure of the compounds, listed in Table 1.

Earlier, several researchers have reported various bioactive polyphenols in SSD [17,40,44,45], in which flavonoids and proanthocyanidins (PACs, condensed tannins) were placed in a prominent position. SSD comprises many secondary metabolites, including (-)-sativan, 3′,4′,7-trihydroxyflavone, 3′-hydroxy-8-methoxyvestitol, 7,4′-dihydroxy-8-methoxy-isoflavone, blumenol A, butin, calycosin, dihydrokaempferol, dihydrokaempferol, dihydroquercetin, dihydroquercetin, dulcisflavan, eriodictyol, formononetin, formononetin, genistein, glycoside, isoliquiritigenin, liquiritigenin, liquiritigenin, medicarpin, naringenin, plathymenin, prestegane, protocatechuic acid, protocatechuic acid, and prunetin, and many of these compounds exert antioxidant, anti-inflammatory, anticancer, and antiviral properties [11,13,17,40,46].

SSD-containing PACs are formed by polymerizing monomeric flavan-3-ols (such as (epi)-catechin (C), afzelechin (FC), or gallocatechin (GC)) as structural units through C–C bonds and/or C–O–C bonds, resulting in condensed tannins [47,48]. Depending on the degree of polymerization (DP) and binding to monomeric flavan-3-ols, they are categorized into monomers, dimers, and oligomers. The DP values are between 3~5, 5~10, and above 10 based on the monomeric flavan-3-ols label, indicating lower, medium, and high levels of polymers, respectively [37,47]. Based on the monomeric structures, procyanidins are called catechin and epicatechin. When afzelechin or gallocatechin are present, they are designated as propelargonidins or prodelphinidins, respectively, and belong to the PAC subfamilies [34,47]. 

**Figure 2:**
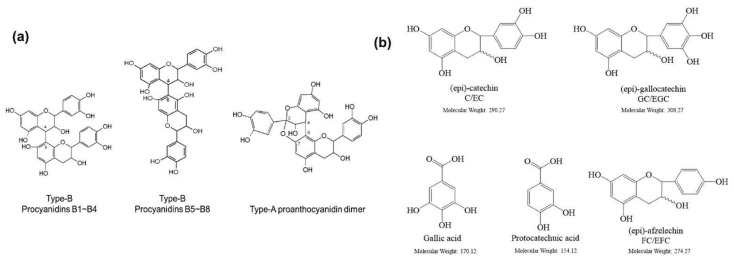
(**a**) Structure of Type-A and Type-B proanthocyanidin dimers. (**b**) Structure of esterified monomers and acids.

Recently, the ((U)HPLC-MS/MS(n)) method has been employed to identify and characterize the chemical constituents of SSD [34,37,38,49,50]. Monomers are mostly linked through C_4_–C_8_ or C_4_–C_6_ linkages, termed B-type linkages. When an additional ether linkage is attached to C_2_–O_7_ bonds, the compounds are named A-type PACs (Figure 2a). Sometimes, an A-type bind with a C_2_–O_5_ linkage is also found. Different structural isomers exist in PACs due to the arrangement of the stereochemical structure. Gallic acid and protocatechuic acid can chemically react with phenolic hydroxyl groups to form esters. In addition, they react with glycosyl and cinnamyl to form glycosides, resulting in more complicated and varied PAC structures [47,48]. The structures of esterified monomers and acids are illustrated in Figure 2b. These esterified monomers and acids of SSD have potential antioxidant, anticancer, and LDH-A inhibitory activity [37], and broad-spectrum viral entry inhibitory activity against various respiratory viral infections viz., SARS-CoV-2, SARS-CoV-1, HIV-1ADA, HIV-1HXB2, H5N1, and Indiana vesiculovirus [51].

## 3. Pharmacological Activity of SSD

Polyphenol-rich SSD has several health benefits as a complementary prescription and is employed in several formulations used for their antioxidant, antidiabetic, anti-inflammatory, anticancer, neuroprotective, hematopoietic, and anti-microbial properties. Supplementary Tables S1 and S2 present comprehensive data on the doses, route of injection, model, positive controls, mechanism of action, and additional pharmacological outcomes, and these data based on research conducted in vitro and in vivo.

### 3.1. Antioxidant Activity

SSD has excellent antioxidant properties due to its phenolic acids, flavonoids, lignans, tannins, and other polyphenols. Principally, catechin, epicatechin, formononetin, gallic acid, syringic acid, and vanillic acid have been described to account for the antioxidant properties of SSD. These antioxidant activities are principally due to their reducing capacity, in which they scavenge or neutralize free radicals and reduce lipid peroxidation [6,7,8]. Furthermore, polyphenols actively involve the chelation of metal ions, causing weakening/cessation of the oxidative process [2,52]. SSD-containing polyphenols exhibited the highest total antioxidant capacity as determined by several in vitro techniques [16,52,53,54,55,56]. SSD exhibited noteworthy neuroprotective benefits on hydrogen peroxide (H_2_O_2_)-induced cell death in PC12 cells [14]. Porcine circovirus 2 (PCV2) infection generates a large volume of nitric oxide (NO), reactive oxygen species (ROS), glutathione disulfide (GSSG), xanthine oxidase (XOD), and myeloperoxidase (MPO) activities, and a severe decrease in the activities of GSH and SOD in RAW264.7 cells. However, treatment with SSD attenuates PCV2-induced oxidative stress markers and enhances the actions of the antioxidant enzymes [54,55]. Animal studies have also confirmed that SSD possesses the highest antioxidant capacity, as determined by various biochemical parameters including thiobarbituric acid reactive substances (TBARS), catalase (CAT), hydroperoxides, glutathione (GSH), glutathione S-transferase (GST), superoxide dismutase (SOD), glutathione reductase (GR), and glutathione peroxidase (GPx) [54,55]. Earlier, one in vivo experiment revealed that SSD treatment reduces malondialdehyde (MDA) and nitric oxide (NO) levels and increases the activities of SOD and GPX in the cerebral ischemia rat brain [57]. Likewise, SSD and its active compound, catechin, restore irradiation-induced hematological parameters, significantly increasing serum SOD, GPX activity, and lower MDA levels and thereby improving the thymus and spleen index [58,59]. Cell lines and animal studies of SSD exercising antioxidant activity are listed in Supplementary Tables S1 and S2.

### 3.2. Antidiabetic Activity

Diabetes is connected with the risk for oxidative damage that may cause protein glycation in tissues and the oxidation of glucose, generating free radicals such as hydroxyl radicals, hydrogen peroxides, and protein-reactive ketoaldehydes, along with a consequential increase in lipid peroxidation, which results in DNA damage [60,61]. Additionally, the generation of advanced glycation end-products triggers NF-κB and its several downstream target genes, resulting in a high amount of NO production that is thought to be a stimulator of β-cell destruction in the pancreas [62]. Therefore, antioxidants can counter oxidative stress and have favorable implications for the management of diabetes. Studies have reported that SSD has a potential antioxidant and antidiabetic effect against glucotoxicity and STZ-induced diabetes in animal models [63,64]. Previously, in vitro and rodent model studies have shown that extract of SSD significantly increased glucose uptake and increased GLUT4 expression in skeletal muscle through the downstream regulation of AKT and AMPK pathways in C2C12 cells and STZ-exposed diabetic mice. Besides, SSD significantly improved the antioxidant enzymes and mitigated the expression of gluconeogenic enzymes in the STZ-induced Type 2 diabetes mice model [63]. Other in vitro anti-diabetic studies have demonstrated a greater inhibition of α-amylase and α-glucosidase that was triggered by SSD due to its rich phenolic compounds [64]. Another in vivo study also confirmed that SSD has a potential anti-hyperglycemic effect by preventing glucotoxicity and diabetes-related renal injury. It markedly reduced serum triglycerides, free fatty acids, LDL cholesterol levels, and histopathological alterations through the Nrf2 pathway [63,64]. SSD inhibited diabetes-related dyslipidemia, advanced glycation end-products, and the ratio of urinary albumin/creatinine in db/db mice [64]. The ethanolic extract of SSD contains enormous quantities of hydrophobic components, including, quinones, steroids, and procyanidins [38], which have potential antiglycation and antidiabetic activities [9,65,66]. In vitro and rodent studies of SSD exerting anti-hyperglycemic activities are depicted in Supplementary Tables S1 and S2.

### 3.3. Anti-Inflammatory Activity

It has been discovered that a number of inflammatory reactions are triggered by mucosal injury, microbial infection, and oxidative stress, leading to the identification of pathophysiological mechanisms that are to detect noxious agents infecting the host, such as lipopolysaccharide (LPS) [9,61]. LPS is an antigen of the bacterial cell, which induces macrophage activation, causing excessive NO synthesis, inflammatory mediators, prostaglandins, TNF-α, and several pro-inflammatory cytokines [67,68]. A possible method for treating inflammatory diseases is to attenuate the production of these inflammatory mediators. 

With mounting evidence from in vitro and animal studies, SSD exhibits a promising efficacy as an anti-inflammatory drug. Aqueous and ethanolic extracts of SSD show a significant effect on the inhibition of NO and TNF-α production [16]. More than dozens of bioactive compounds isolated from SSD such as (+)-epipinoresinol, (+)-pinoresinol, 2,6-dimethoxy-1,4-benzoquinone, 3-methoxydaidzein, 8-O-methylretusin, biochanin A, butesuperin A, calycosin, daidzin, formononetin, genistein, isoliquiritigenin, liquiritigenin, maackiain, odoratin, and ononin have been screened for their anti-inflammatory efficacy [69]. Furthermore, Spasuberol A, B, and C have also been identified and characterized from SSD and have been reported as anti-inflammatory agents, which was determined by decreasing NO production in RAW264.7 macrophages stimulated by LPS [42].

Polyphenol-rich extracts of SSD are potent anti-inflammatory agents against COX-1/2, phospholipase A2, 5-lipoxygenase (LOX), and 12-LOX [70]. Another study shows that flavonoids such as liquiritigenin, isoliquiritigenin, catechin, butin, 3′,4′,7-trihydroxyflavone, plathymenin, 2,6-dimethoxy-1,4-benzoquinone, genistein spasuberol C, and gallocatechin have been extracted from SSD, which are potent inhibitors of 5-LOX and NO production and reduce some pro-inflammatory cytokines, including TNF-α, iNOS, and COX-2 [42,71]. Studies reported that liquiritigenin blocks NF-κB and MAPK pathways and inhibits the production of IL-2, CD69, CD40L, and CD25 in T cell activation [72]. In an atopic dermatitis clinical model, oral administration of liquiritigenin reduced the onset of diseases such as ear thickness, IgE levels, the thickness of the dermis, and epidermis; thus, liquiritigenin manifests a systemic anti-atopic effect and exhibits a therapeutic effect for T cell-mediated disorders [72]. 

Twenty-five bioactive compounds from SSD displayed anti-inflammatory properties against the LPS-stimulated overproduction of NO in RAW264.7 cells. Importantly, isoflavones from SSD appear to be key components of anti-inflammatory products. After oral treatment with SSD in rats, the pharmacokinetic traits of these twenty-five compounds in the plasma of rats were quantified by UFLC-MS/MS. This investigation, furthermore, offers reference values for the clinical studies and pharmacological activities of SSD [73]. In vitro and in animal studies of SSD exerting anti-inflammatory efficacies are depicted in Supplementary Tables S1 and S2.

### 3.4. Neuroprotective Activity

Apoptotic cell death in neuronal cells plays a key role in the control of several neurological disorders, viz., Parkinsonism, Alzheimer’s, Huntington’s, and ischemic stroke. Studies show that SSD has therapeutic potential for neurological-disorder-related cell death or ischemic stroke [74]. Study results showed that SSD prevented nerve injury and etoposide-induced neurotoxicity in vitro and focal transient ischaemic stroke MCAO-reperfusion injury in rats. SSD significantly improved apoptotic phenotypes in SH-SY5Y cells after treatment with etoposide-induced cell death. In an animal study, SSD improved neuronal survival and BDNF levels by apoptosis in the ipsilateral cortex, lowering glial cell activation and JNK/p38 MAPK activation [74]. 

### 3.5. Hematopoietic Activity

SSD has been employed to heal blood stasis syndrome by impeding platelet aggregation, pro-angiogenesis, and provoking hematopoiesis since time immemorial. By stimulating hematopoiesis and erythroid cells, catechin in SSD may inhibit the disease-related blood stasis syndrome [75,76]. Clinical studies have further established that a decoction of SSD stimulates hematopoiesis and restores the marrow microenvironment [77,78]. SSD exerts the most powerful inhibitor of hepcidin antimicrobial peptide (HAMP) [79], which is primarily involved in the maintenance of iron homeostasis and anemia of chronic disease and hemochromatosis. The animal study, associated with the supplementation of SSD, showed the most potent inhibitory effect on HAMP expression by reducing hepatic iron concentration and increasing serum iron load [79]. SSD promotes hematopoiesis induction by initiating stroma cells in the hematopoietic settings and muscle tissue resulting in HGF [80]. SSD stem subfractions stimulate the proliferation of hemopoietic progenitor cells in a dose- and time-dependent manner in a bone marrow-depression model [80]. Both in-vitro and animal assays reported that catechin extracted from SSD promotes the stimulation of hematopoiesis through various cytokines and chemokines, allowing for a normal cell cycle in bone marrow stem cells and exiting the ‘G1-phase-block’ in marrow-depressed mice [75,81]. In vitro and in vivo studies of SSD performing the stimulation of hematopoietic activities are represented in Supplementary Tables S1 and S2.

### 3.6. Antimicrobial Activity

Various extracts of SSD have shown excellent antimicrobial properties. As of now, SSD has been recognized to prevent the growth of bacteria (*Staphylococcus aureus* and *S. mutans*) and viruses (HIV Type 1, HCV, Coxsackievirus B3) [82,83,84] Among its crude extracts, 7-hydroxy-6-methoxyflavanone, EGCG, EGC, and formononetin have been reported to have notable anti-microbial properties. Methanolic extracts of SSD showed significant inhibition (>90%) effects on HIV Type 1 [82] and inhibit all three stages of the virus’s life cycle in Coxsackievirus B3 [83], which has been determined through the lessening of infection titers in the tainted cardiac cells of rats. Furthermore, SSD alleviated the Coxsackievirus B3-induced viral myocarditis and reduced mortality in animal models [11]. SSD is a significant anti-hepatitis C viral agent. Remarkably, its crude extracts exhibited better antiviral activity than that of pure compounds such as EGCG and EGC [85]. SSD is an established and effective anti-multidrug-resistant *Staphylococcus aureus* (MDRS) agent with comparatively mild cytotoxicity [84]. Moreover, SSD, containing 7-hydroxy-6-methoxyflavanone and formononetin, shows considerable inhibitory activity by preventing *S. mutans* SrtA without any evidence of cytotoxicity [35,86]. In vitro and animal studies of SSD employing antimicrobial activities are summarized in Supplementary Tables S1 and S2.

### 3.7. Other Activities

SSD has been reported to show potential bioactivities such as anti-platelet aggregation, cytochrome P450 inhibition, anti-osteoclastogenesis, and kinetoplast DNA decatenation [87,88]. Previously, studies exhibited that the ethanolic extracts of SSD significantly reduce platelet aggregation and inhibit fibrinogen formation by interacting with the production of thromboxane A. This is due to the activation of GP IIb/IIIa receptor. Thus, oral administration of SSD significantly prevents the mortality of mice by thromboembolism [89]. Procyanidin B4 obtained from SSD has been reported to be a potent inhibitor of DNA-topoisomerase-II-mediated KDNA decatenation [90]. SSD is recognized to have significant preventive effects on cytochrome P450 that involve potential drug interactions [88]. Based on molecular modeling and multivariate chemometric analysis, these drug interactions can be attributed to the occurrence of polyphenols in SSD [87]. SSD can be used as medicine in the treatment of osteoporosis and rheumatoid arthritis. Preceding studies suggested that crude extracts of SSD inhibited the conversion of RANKL-stimulated osteoclast precursors into osteoclasts by inhibiting MMP, c-Fos, NFATc1, and TIMP [91,92]. In vitro and in vivo studies of SSD exerting other activities are summarized in Supplementary Tables S1 and S2.

## 4. Anticancer Activity of SSD

There has been evidence that SSD plays a positive role in the treatment of leiomyoma, breast cancer, glioblastoma, and leukemia. The anti-breast cancer effect of SSD has attracted the most attention among researchers. SSD has potential anticancer effects through apoptosis and pyroptosis induction, cell-cycle arrest, estrogen receptor hypoactivity, proteasome inhibition, anti-mutation, and ROS regulation [13,24,27,40,50,93,94]. Surprisingly, unlike anti-glioblastoma, SSD’s anti-breast cancer and anti-myeloma efficacy are reliant on ROS induction. In an earlier clinical cohort study, it was demonstrated that SSD has prospective benefits for patients with acute myeloid leukemia [95]. The chloroform and ethyl acetate subfractions of SSD administration were reported to be potent by preventing leiomyoma and reducing the expression level of TGF-beta receptor 2 [96]. A dose-dependent manner of SSD treatment exerted cytotoxicity in the myeloid-originated hematological cancer cell lines U266 and U937, which upregulated apoptosis-related proteins (PARP, procaspase-3, and Bax) and ER stress-related proteins (p-ATF2 and CHOP). Furthermore, SSD inhibited onco-miRNA (miR657) targeting the ER stress signal pathway [93]. Interestingly, KEGG and GO analysis interpreted that SSD could attenuate metastasis in the lung primarily by mediating oxidative stress, AGE-RAGE signaling, and microRNAs [97,98]. Similarly, Network pharmacology analysis revealed that SSD exhibited an anti-ovarian cancer efficacy by activating the key proteins GSK-3β, Bcl-2, and Bax [99].

SSD has been reported to inhibit cervical cancer cell growth in earlier studies [100]. The tannin from SSD has been demonstrated to prevent the conversion of cells from G0/G1 to the S phase and G2/M phase. This inhibits cell proliferation by impeding gene and protein synthesis. SSD has therefore been demonstrated to exhibit inhibitors of cancer and proliferative characteristics in HeLa cervical cancer cells [101]. Furthermore, SSD mediates related circRNAs to promote apoptosis and impede proliferation in HeLa cells [102]. Similarly, the extract of SSD showed potential cytotoxic efficacy on the human lung cancer cell line, A549, which increases the S-phase and decreases the G0/G1 and G2/M phase and thus promotes non-apoptotic programmed cell death [103]. Furthermore, the methylene chloride fraction of SSD in a dose-dependent manner presented potential toxicity by killing human monocyte leukemia U937 cells, which triggered caspase-3 expression and cleaved PARP [104]. Cao et al. [105] found that compound 1802 obtained from an aqueous extract of SSD showed an inhibiting effect on tumor growth in a dose-dependent manner. According to another study, SSD extracts were reported to have varying degrees of inhibitory activity against human osteosarcoma Saos-2 cells when ethanol, ethyl acetate, and n-butanol extracts were used [24].

Previously, studies found that EGCG, a flavanol in SSD, promotes apoptosis in the head, neck, and colorectal cancer [106,107]. Furthermore, the anticancer effect of gallic acid obtained in SSD was present in lung and prostate cancer [108,109]. Moreover, our lab previously reported a novel function of isoliquiritigenin, the active principle from SSD, as a natural inhibitor of autophagy-related miR-25 that promoted autophagy, chemosensitization, and cell-cycle arrest in drug-resistant MCF-7/ADR BC cells. Thus, isoliquiritigenin acts as a natural autophagy inducer to enhance BC chemosensitivity by targeting ULK1 [110]. Furthermore, our laboratory has confirmed that Isoliquiritigenin is synergistic with chemotherapeutic drugs in preventing the proliferation of BC cells and the formation of colonies [111]. Treatment with isoliquiritigenin inhibits tumor growth, EMT, and metastasis in nude mice by decreasing miR-200c, miR-374a, c-Jun expression and increasing PTEN expression, thus preventing TNBC [112,113]. Additionally, in vivo study also reveals that Isoliquiritigenin could chemosensitize BC stem cells via the GRP78/β-catenin/ABCG2 pathway [114,115]. 

Isoliquiritigenin was investigated as a natural demethylation agent that targets WIF1 signaling to prevent BC. Treatment with Isoliquiritigenin reduced in vivo BC progression, escorted by decreased BC stem cell populations; thus, Isoliquiritigenin prevents mammary carcinogenesis [116]. However, the bioavailability and delivery characteristics of Isoliquiritigenin are poor. In order to increase the therapeutic results of Isoliquiritigenin, our lab constructed Isoliquiritigenin with iRGD modified lipid–polymer hybrid nanoparticles (ISL–iRGD NPs), which exhibited significant cytotoxicity and apoptosis on BC cells. Furthermore, in vivo results also confirmed the prolonged accumulation of novel ISL–iRGD NPs in the tumor tissue and blood circulation, which enhanced the efficacy of tumor-growth inhibition in 4T1-BC mice models [117]. The SSD stems were shown to suppress BC both in vitro and in rodent models through preventing cell-cycle progression at the G2/M phase. This is done through DNA damage, activation of Chk1/Chk2, and the proapoptotic process, which can be key factors contributing to BC suppression [94]. SSDs have been studied for their potential pre-clinical, clinical, or antitumorigenic effects in BC [13,27,29,40,118]. In vitro studies on SSD have been investigated by inhibiting several BC models that endure downregulating cell signaling pathways. This has effects on the induction of apoptosis, cell growth, cell-cycle arrest, reduction of angiogenesis, cessation of invasion, and metastasis [27,58,80,94,118]. It effectively inhibits BC in a dose-dependent manner. In earlier BC animal model research, it was shown that SSD had a better effect than docetaxel treatment. No apparent body weight changes or blood toxicity was detected in SSD-treated animals. Additionally, according to recent laboratory data, SSD exhibits anti-TNBC characteristics that depend on ROS-induced noncanonical inflammasome pyroptosis [119].

Lactate dehydrogenase A (LDH-A) is a recognized innovative target for cancer therapy and the development of LDH-A inhibitors from bioactive compounds derived from herbs has been gaining interest in drug development [118,120,121]. Our lab previously investigated the role of LDH-A reduction in preventing BC proliferation, increasing cellular oxidative stress, and promoting apoptosis in the mitochondrial pathway, both in vitro and in xenografted mice bearing MDA-MB-231 [121]. Furthermore, LDH-A expression is reported to be associated with tumor size and other clinicopathological factors [121]. In vitro and animal model studies demonstrate that SSD containing EGC induces apoptosis in BC cells by inhibiting LDH-A activity. Additionally, the administration of EGC promotes Hsp90 dissociation from HIF-1 and thus increases the degradation of HIF-1 [118]. Additionally, the vine extracts of SSD inhibit proliferation and migration through the MAPK PI3K/AKT signaling pathway, as well as stimulate apoptosis and prevent cells from cell cycling in the MCF-7 cell line [27].

The chemical constituents of SSD have massive pharmacological effects and clinical applications. SSD contains the analogue of isoliquiritigenin that is used for the prevention of BC [29]. Cyclization of OH-2′ chalcones or α, β-unsaturated isoliquiritigenin significantly induced cytotoxicity in BC cell lines. Likewise, 3′,4′,5′,4′′-tetramethoxychalcone is also known as a promising cytotoxic analogue against BC [29]. Interestingly, 3S)-7-hydroxy-8,2′,4′-trimethoxyisoflavane and (3S)-7-hydroxy-8,2′-dimethoxy-4′,5′-methylenedioxyisoflavane in SSD inhibit cell growth and the viability of MCF-7 cells. Also, sativan extracted from SSD is highly hazardous to both MCF-7, 4T1, and MDA-MB-231 cell lines, indicating that SSD is a potential candidate for the inhibition of TNBC cells [29]. Moreover, sativan, another active compound extracted from SSD, has been described to improve the expression of Bax and reduce the protein expressions of Bcl-2 and PD-L1, inhibiting the invasion and migration of TNBC cells by the upregulation of miR-200c [46].

SSD significantly inhibits C6 glioma cell growth through its antioxidant properties in glioblastoma. Moreover, SSD has an anti-glioblastoma effect that inhibits mitochondrial membrane depolarization, upregulating the expressions of p53, p21, STAT3, and E2F1, and downregulating Bcl-2 and cell-cycle arrest [122]. SSD plays a progressive function in the prevention of myeloma and BC through the induction of apoptosis and pyroptosis through the activation of PARP, GSDME, and CHOP [93,94,119]. Platelet aggregation caused by tumor cells is the underlying mechanism for high levels of circulatory tumor cells, which is key to metastasis. Ethyl acetate fractions of SSD significantly reduced the invasion and metastasis of colorectal cancer by blocking the release of platelet-derived PDGF-B and EMT, thus preventing tumor cell-induced platelet aggregation in the colon [123]. Similarly, the ethyl acetate fraction of SSD inhibited the platelet aggregation induced by B16BL6 melanoma cells through the downregulation of urokinase-type plasminogen activator, resulting in the suppression of tumor cell invasion in the skin [124]. SSD exerted a significant activity on the prevention of BC metastasis and increased the rate of survival in 4T1 cells injected mice [125].

Methanolic extracts of SSD contain several bioactive compounds, including isoliquiritigenin, genistein, 7-hydroxyflavanone, liquiritigenin, daidzein, medicarpin, and formononetin, which have significant inhibitory activity on the human 20S proteasome [69]. Various proteasome inhibitors exert anti-cancer activity in vivo and significantly induce apoptosis in cancer cells in vitro [2,8,65], and SSD and its active compounds are potential proteasome inhibitors [69]. The prevention of *N*-nitrosamine-induced DNA damage is a key factor for cancer chemoprevention. It has been reported that SSD containing the bioactive compounds genistein, isoliquiritigenin, medicarpin, and naringenin has antigenotoxic effects by preventing production of the carcinogenic *N*-methyl-*N*-nitrosourea [126]. It seems that flavonoids have hydroxyl radical-scavenging capacity that is connected to their antimutagenic properties [126]. 7,4′-dihydroxy-8,2′,3′-trimethoxyisoflavan extracted from SSD has significant cytotoxicity in preventing human cancer cell lines, viz., HL-60, SMMC-7721, SW-480 MCF-7, and A-549 [41]. The ethanolic extract of SSD contains four novel isoflavanes, which exert potential cytotoxicity activities in MCF-7 and MDA-MB-231 BC cell lines. [40]. In vitro and animal model studies on the chemopreventive and anti-cancer effects of SSD are depicted in Table 2 and Table 3.

## 5. Mechanism of Anticancer Activity of SSD

SSD and its active constituents elicit their anticancer activities through several distinct pathways. The anti-tumorigenic effects of SSD on the breast captivate much more attention in the research community, so we focus exclusively on anti-breast cancer activities and their underlying molecular mechanisms. SSD promotes anti-BC effects by networking with membrane-bound ER, inhibiting the activities of G-protein mediated signaling, protein kinases, receptors of growth factor, and their distinct signaling pathways. In this context, SSD and its compounds inhibit cancer cell proliferation through two downstream signaling pathways, viz., RAS/Raf/MAPK and PI3K/Akt/mTOR.

### 5.1. RAS/Raf/MAPK Signaling

**Figure 3:**
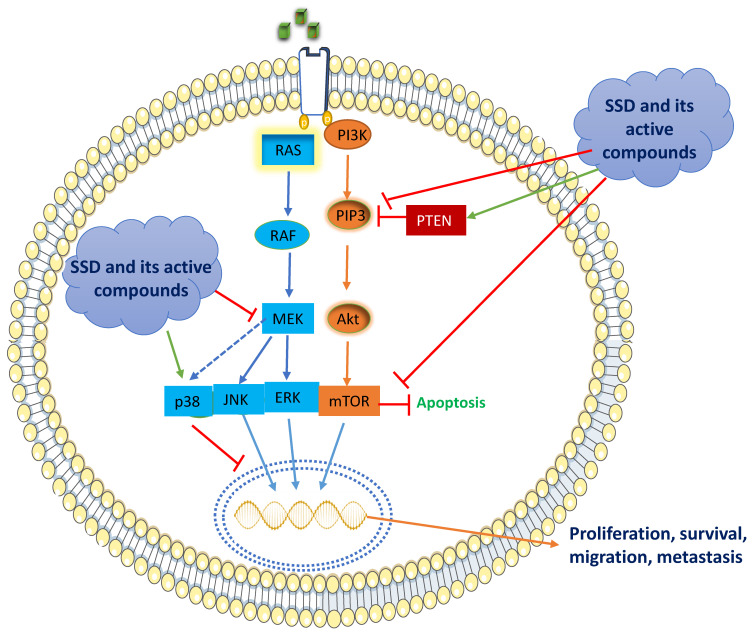
The role of SSD and its active constituents in inhibiting cell proliferation, survival, migration, and metastasis through the RAS/Raf/MAPK and PI3K/Akt/mTOR signaling pathways.

MAPK is an active signaling cascade pathway in BC that encompasses numerous key pathways involving a phosphorylation mechanism that has a primary function in tumorigenesis. Signaling pathways are altered in cancer, leading to an aberrant stimulation of tyrosine kinases, which leads to an increase in RAS/RAF gene expression, providing uncontrolled cell proliferation, survival, invasion, and metastasis—hallmarks of cancer [143]. The downstream cascade signals, RAS/RAF/MAPK, have a key function in cell survival and the development of BC (Figure 3). MAPK pathway generally comprises the tumor suppressor gene, *p38*, ERK 1/2, and JNK 1/2 in normal breast cells [144]. However, activation and upregulation of ERK 1/2 and JNK 1/2 occur in BC cells, which promotes transcription activation of genes related to uncontrolled cell growth [145]. SSD and its active constituents provoke their anti-BC effects by networking and inhibiting the downstream signaling pathways of RAS/RAF/MAPK.

For example, calycosin treatment of MCF-7 and MDA-MB-231 BC cells caused an increased phosphorylation of p38 in a dose-dependent manner (25–100 μM) [146]. Earlier, our lab also reported that Isoliquiritigenin (5–20 μM), another active constituent of SSD, decreased the phosphorylation of JNK 1/2 and MAPK in MCF-7 cells, thereby concentrating the inhibitory effect of MAPK signaling and thus suppressing BC development [147]. MAPK signaling pathway is considerably reduced by treatment with delphinidin in a dose-responsive manner (12.5–100 μg/mL) in SKBR3, BT474, HCC1806, MCF7, MDA231, MDA468, and MDA453 BC cell lines [148]. EGCG, a bioactive catechin present in SSD, has been shown to have anticancer activity against human BC MCF-7, MCF-7TAM, and MDA-MB-231 cells. This compound significantly reduced the cell proliferation and migration of BC cells by the expression of p-ERK1/2, p-p38, and p-JNK in a dose- and time-responsive manner [149]. EGCG was administered to experimental MCF-7 xenografted mice at 5 mg/kg, i.p., for 14 days, which inhibited the phosphorylation of ERK, JNK, and p38 MAPK [150]. Formononetin, an active ingredient of SSD, has been reported to inhibit MCF-7 BC cells through downregulating the expression of phosphorylated ERK [151]. Another study showed that formononetin in combination with calycosin impaired cell growth and promoted apoptosis in MCF-7, and T-47D BC cells by decreasing IGF-1R, miR-375, and p-ERK1/2 [152]. Similarly, genistein (5, 10, 20 μM) treatment significantly decreased MAPK signaling by decreasing the expression of MEK5, ERK5, and p-ERK5 in MDA-MB-231 BC cells [153]. Overall, pre-clinical results indicate that SSD and its active constituents inhibit BC development via preventing ERK1/2 phosphorylation, activating p38 phosphorylation, and modulating the expression of the MAPK pathway.

### 5.2. PI3K/Akt/mTOR Signaling

The remarkable downstream signaling of PI3K/Akt/mTOR is frequently engaged in most of the BC types, accounting for over 70% [154,155,156,157]. In turn, PI3K stimulates the conversion of PIP2 to PIP3, which phosphorylates protein kinase B, Akt, which in turn activates the serine/threonine kinase, mTOR [158,159]. The cascade signaling of PI3K/Akt/mTOR is a crucial key step in the cell cycle, tumor development, and survival [155]. However, the dephosphorylation of PIP3 to PIP2 activates Akt by an enzyme, PTEN, which is a well-recognized tumor-suppressor protein. (Figure 3) [156]. Activation of the PI3K/Akt pathway occurs via insulin-like growth factor 1 receptor (IGF-1R), thereby phosphorylating IGF-1R and resulting in the recruitment of Akt [160]. 

SSD and its active constituents exert their anti-cancer effects by networking with complex transcription factors, PI3K/Akt/mTOR. *SSD* comprises calycosin daidzein, formononetin, and genistein with a dose range of 80–320 μg/mL that inhibits the protein expressions of PI3K/Akt/mTOR in MCF-7 cells [27]. Resveratrol and genistein in the doses of 0.1, 1, 5, 10, 100, and 1000 nM significantly elevated PTEN expression (PI3K/Akt inhibitor) in MCF-7 and MDA-MB-435 BC cells [161]. The treatment of calycosin (25–100 μM) and formononetin (20–80 μM) inhibited PI3K/Akt signaling in T47D and MCF-7 cells by reducing the activation of IGF-1R protein levels and resulting in the inhibition of Akt phosphorylation [84,130]. Similarly, the treatment of formononetin (10–40 μM) decreased the expression of p-PI3K/p-Akt in TNBC cells [162]. Another investigation demonstrated that an analog of resveratrol, MR-3 (10, 20 μM), attenuated PI3K/Akt signaling in MCF-7 cells by preventing the phosphorylation of Akt and inhibition of GSK-3β [163]. SSD contains another bioactive compound, baicalein, which is demonstrated to have an anti-BC effect through the downregulation of the expression of p-AKT, p-mTOR, NF-κB, and p-IκB and the upregulation of IκB in MCF-7 and MDA-MB-231 cells [92]. Altogether, SSD and its active constituents inhibit the PI3K/Akt pathway by inhibiting IGF-1R, phosphorylating Akt, and enhancing the inhibitory activity of PTEN.

## 6. Clinical Studies

There are no known well-designed clinical studies available related to SSD on human cancers. According to clinicaltrials.gov, a study is currently underway focused on the renal protective effects of Yi-Qi-Qing-Jie formula (YQF), containing SSD, that aims to prevent end-stage kidney disease in patients (NCT03418779). This ongoing clinical trial is a single-center, prospective, double-blind, placebo-controlled trial aiming to assess the renal protection and reduction of severe treatment-related adverse events of YQF (phase II) combined therapy compared with immunosuppression monotherapy (phase III) in high-risk IgA nephropathy patients. 

In addition, SSD formulations have a variety of pharmacological effects and are used in TCM to prevent many diseases. Several clinical trials confirm that Chinese herbal medicine has been employed for the treatment of chemotherapy-induced leukopenia. SSD is one of the components that plays a significant role in the treatment of patients with leukopenia, which includes Husui decoction, Qijing Shengbai granules, modified Sancai Fengsui decoctions, Sanhuang Sanxian decoctions, and Bazhen decoctions [164,165,166,167]. An exclusive invention in the field of medicine formulation allows for the treatment of thrombocytopenia after radio–chemotherapy, using a decoction of Chinese medicinal herbs. The decoction contains SSD, which provides the effects of nourishing the liver and kidney, nourishing Yin and blood, and cooling blood to stop bleeding. No toxic or side effects have been reported in patients with thrombocytopenia [168]. Thus, SSD has no negative effects or complications.Tongmai Yangxin Pill is a Chinese medicine derived from two traditional Chinese prescriptions, Shenmai Yin and Zhigancao Decoction, which are a combination of eleven Chinese traditional herbs. Among them, SSD is one of the ingredients that have a significant role in the treatment of patients with coronary heart diseases and inflammatory diseases [169]. It has been used to treat patients with severe aplastic anemia [170]. According to a nationwide population-based cohort study, SSD is the eighth most common herb used in the treatment of patients with chronic myeloid leukemia in adjunctive Chinese herbal medicine [171]. Based on a recent clinical trial, Tongmai Yangxin pills containing SSD reduced apolipoprotein B, endothelin 1, NF-κB, and homocysteine levels in patients with coronary heart disease. Furthermore, this drug reported anti-inflammatory effects by modulating the expression of estrogen receptor 1, as well as inhibiting NF-κB p65, MCP-1, TNF-α, and IL-6 production [169]. A multicenter, double-blind, randomized, placebo-controlled clinical trial was carried out in patients with coronary heart disease, and it delayed the development of heart failure and decreased the rate of cardiac dysfunction [172]. Another clinical study was conducted with Tongmai Yangxin formula on pediatric Graves’ disease patients (younger than 7 years old). When compared with the placebo, administration of a crude formula (150 mL/day) for 60 days greatly diminished irregular glucose tolerance and insulin sensitivity [173]. Since ancient times, Chinese herbal medicine has been used to help treat knee osteoarthritis. Various decoctions composed of SSD have a potential role in the prevention of knee osteoarthritis. Previously, SSD was employed as the main ingredient in Gubi Decoction [174], and Bushen Huoxue Decoction [175], which are being used in the treatment of osteoarthritis. Although Chinese medicine practitioners treat patients with chronic myeloid leukemia with SSD as a herb, well-controlled clinical trials are necessary before using SSD composites to treat cancer in order to demonstrate its efficacy and safety.

## 7. Toxicity Studies

Earlier, acute toxicity studies were performed in our lab to determine the detrimental effects of SSD on rodents that were treated orally as a single or short-term treatment for 24 h [13]. After investigating and comparing low and high doses, it was determined that there were no changes in the rodent’s signs and symptoms and overall health. The acute toxicity was reported in the oral treatment of SSD with an LD50 value of 10 g/kg b.w [13]. No irritation was induced by injecting the SSD into different sites in rabbits. It did not cause an allergic reaction in guinea pigs. Clinical trials also confirmed that no toxic side effects or adverse reactions were found after being treated in patients with thrombocytopenia [168] and leukopenia with lung cancer [166].

## 8. Future Perspectives

SSD and its active constituents have shown remarkable potential in the treatment of various cancers including BC in vitro and in vivo. The function of SSD is to enhance the microenvironment within the body, increase blood circulation, remove harmful substances and maintain the local virtuous cycle. Various investigations have explored a variety of signaling pathways that modulate transcription factors and protein kinases [176]. There were no scientific data available related to SSD on human cancers. Several practical flaws still exist in clinical trials, such as placebo control, sample size, duration, etc. Therefore, large-scale and well-controlled clinical trials are imperative before the use of SSD composites for cancer treatment, in order to verify their effectiveness, adverse effects, and safety. Further, extensive calibration in terms of the techniques used for assessing SSD’s bioavailability, composition, effectiveness, as well as its development processes, quality, safety, and regulatory practices, must be obtained for a favorable SSD in order to meet global standards [177]. As a result, SSD will become more attractive owing to its antioxidant and anti-inflammatory properties that initiate alternative signaling pathways.

## 9. Conclusions

SSD and its active compounds are auspicious medicinal drugs implicated in the treatment of various ailments since time immemorial. SSD comprises distinguished polyphenolic compounds, including flavonoids, chalcone, dihydroflavone, pterocarpan, and phenolic acid. These compounds have potential pharmacological effects, viz., antioxidant, antidiabetic, antimicrobial, hematological, neuroprotective, and anticancer properties. There is significant potential for both in vitro and in vivo studies to combat BC types. Furthermore, SSDs exert their anti-tumor effects through modulating PI3K/Akt/mTOR and Ras/Raf/MAPK pathways, preventing BC development. Cellular and molecular studies have more significantly supported such properties/active constituents, whereas human studies are still lacking. Since in vivo studies do not largely interpret clinical settings, additional human cohort studies are warranted. This will enable us to fully comprehend the effects of SSD on healthcare and disease prevention. Then, prospective far-reaching clinical experiments are necessary to justify the therapeutic effect of SSD. This review will, however, provide a series of treatment options to treat many diseases with negligible side effects.

## Figures and Tables

**Table 1:** Structure of compounds identified in SSD.

Class	Sub-Class	Chemical Name	Structure	References
Carotenoids	Apocarotenoids	Blumenol A	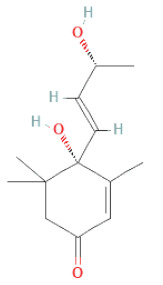	[33]
Coumestans	Coumestans	Medicagol	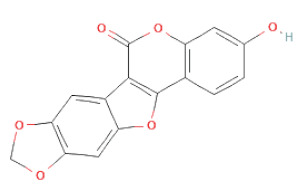	[34]
Flavanoids	Flavonols	Rutin	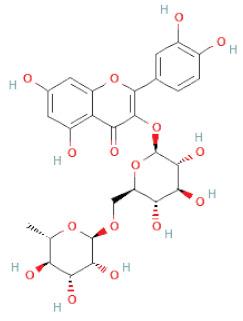	[28]
	Flavanols	Epigallocatechin	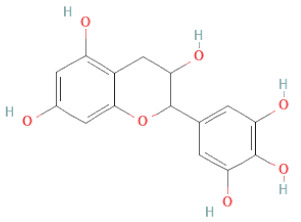	[26,28,34]
		Procyanidin B2	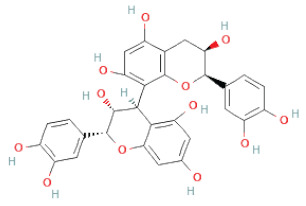	[26,28,35]
		Epicatechin gallate	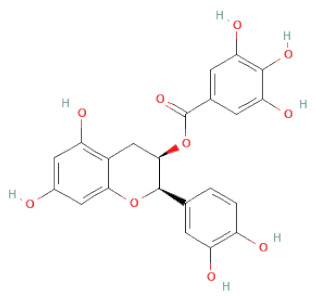	[34,36,37,38]
		Methyl gallate	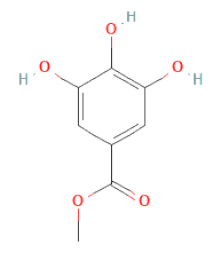	[34,36,37,38]
		Ethyl gallate	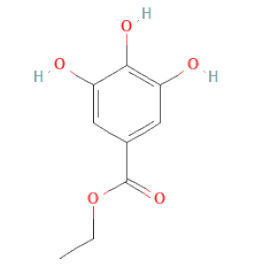	[34,36,37,38]
		3,7-dihydroxy-6-methoxyflavonol	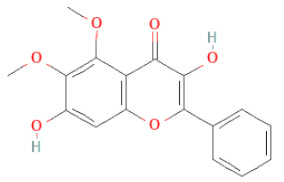	[34,36,37,38]
		(-)-Catechin	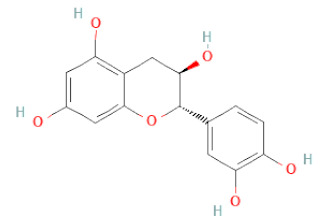	[30]
		(-)-Epiafzelechin	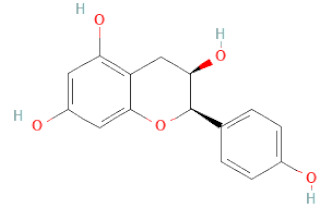	[30]
		(-)-Epicatechin	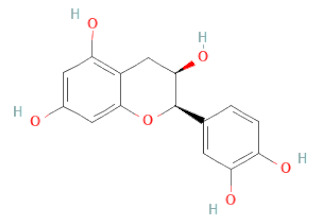	[20,26,27,28,30,34,35,39]
		(-)-Gallocatechin	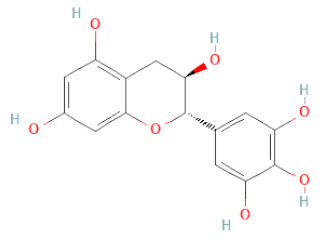	[20,26,28,30,34,39]
		(+)-Catechin	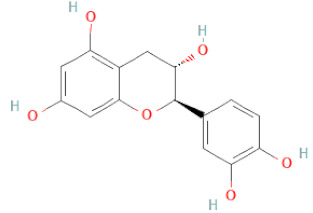	[35]
		(+)-Gallocatechin	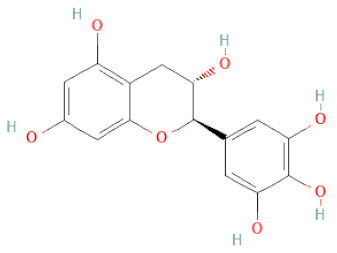	[35]
	Flavanone	Suberectin	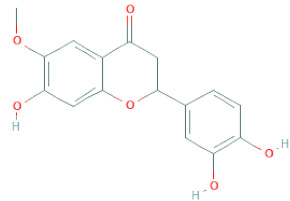	[18]
		Hesperetin	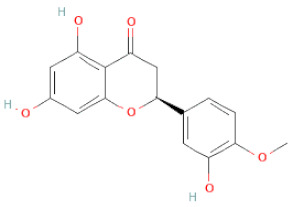	[34]
		Naringenin	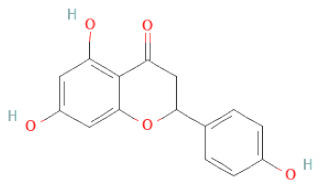	[26,28,33,34,35]
	Flavonols	Kaempferol	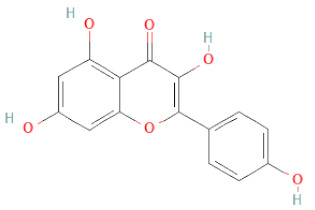	[28]
	Isoflavonoids	Biochanin A	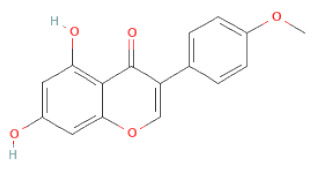	[34,36,37,38]
		2′-Hydroxygenistein	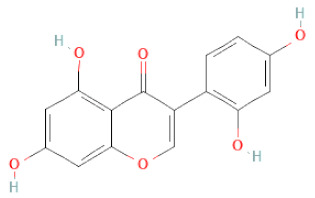	[30]
		3′-Methoxydaidzein	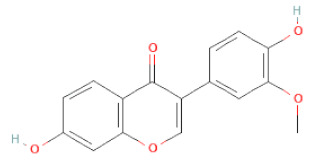	[30]
		5,7-Dihydroxy-4′-Methoxyisoflavone	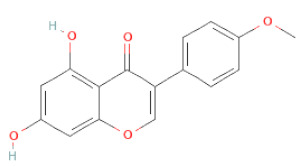	[26,28,30,34,35,40]
		7,2′,4′-Trihydroxy-8,3′-Dimethoxyisoflavan	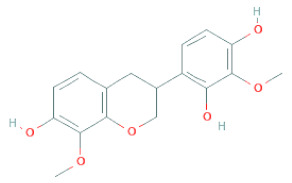	[41]
		7,2′-Dihydroxy-4′,5′-Methylenedioxyisoflavan-4-Ol	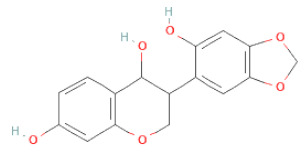	[35]
		7,4′-Dihydroxy-8,2′,3′-Trimethoxyisoflavan	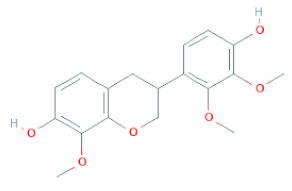	[41]
		7,4′-Dihydroxy-8-Methoxy-Isoflavone	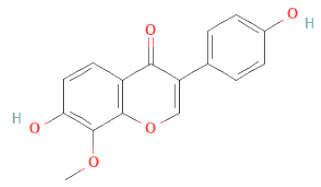	[33]
		Afrormosin	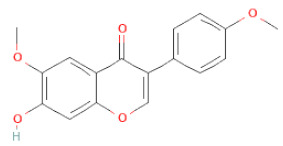	[34,35]
		Butesuperin A	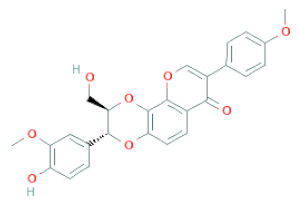	[30]
		Calycosin	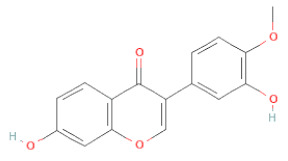	[18,26,27,28,30,34]
		Daidzein	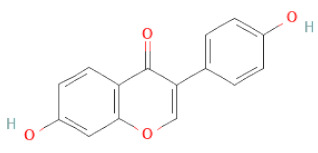	[18,26,27,28,30,34,35,42]
		Daidzin	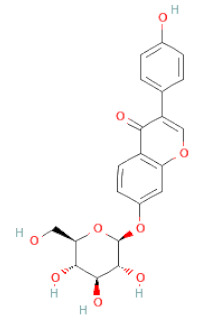	[28,30]
		Dulcisflavan	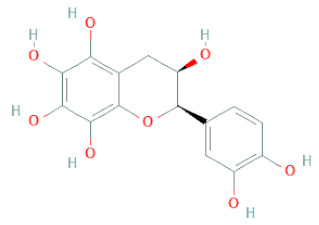	[33]
		Formononetin	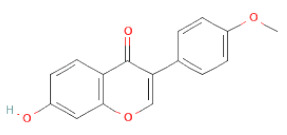	[18,26,27,28,30,34,35,42]
		Genistein	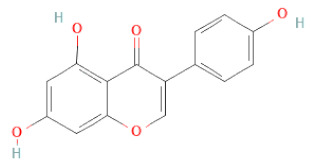	[26,27,28,30,34,35,40,42,43]
		Genistin	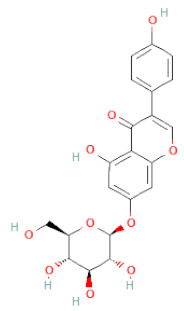	[28,30,42]
		Glycyroside	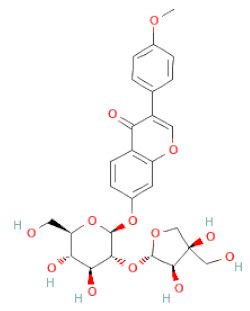	[33]
		Isoliquiritigenin	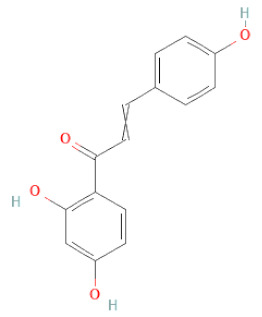	[26,28,30,34,35,42]
		Liquiritigenin	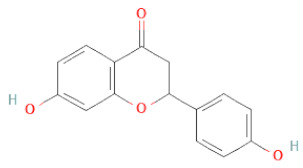	[26,28,30,33,34,35,42]
		Maackiain	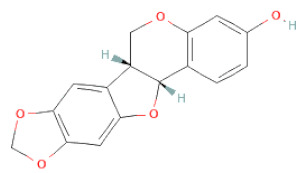	[30,40,43]
		Medicarpin	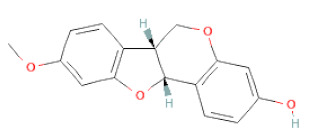	[28,43]
		Ononin	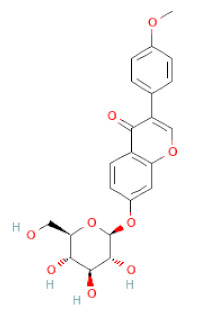	[20,26,28,30,34]
		Prunetin	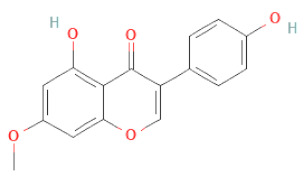	[26,27,28,34]
		Pseudobaptigenin	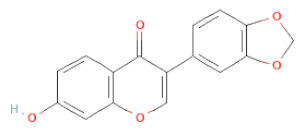	[34,43]
		Puerarin	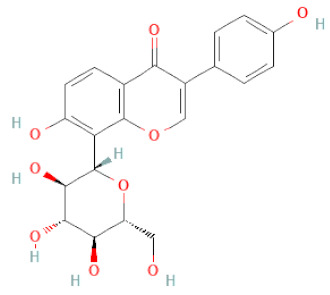	[27]
		Sativan	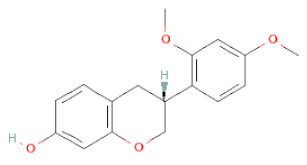	[30,35,43]
	Chalcones	Butein	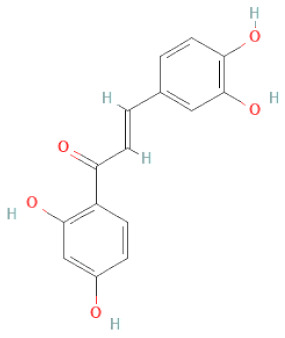	[34]
	Dihydroflavonols	Dihydroquercetin	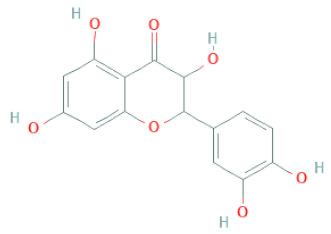	[26,28]
	Flavanone	(2R,3R)-3,7-dihydroxyflavanone	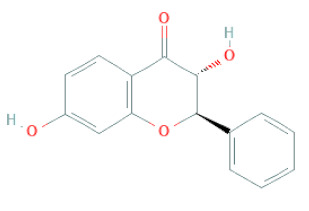	[33]
		(2S)-7-hydroxy-6-methoxy-flavanone	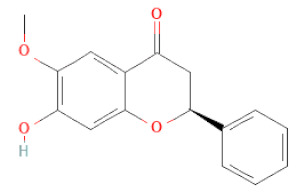	[43]
		(2S,3R)-3,7-dihydroxy-6-methoxy-flavanone	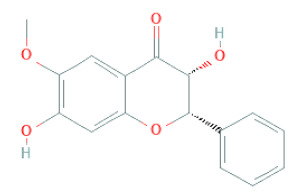	[35]
		7-hydroxyflavanone	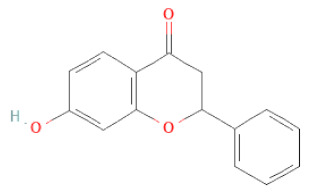	[35]
Lignans	Lignans	(+)-Epipinoresinol	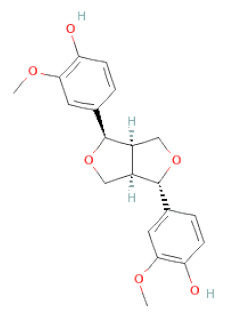	[30]
		(+)-Medioresinol	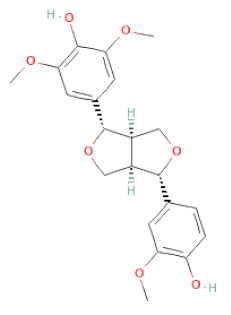	[30,33]
		(+)-Pinoresinol	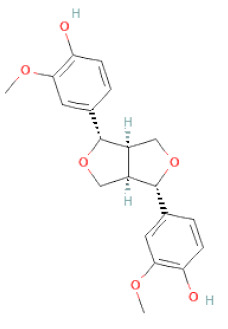	[30]
		(+)-Syringaresinol	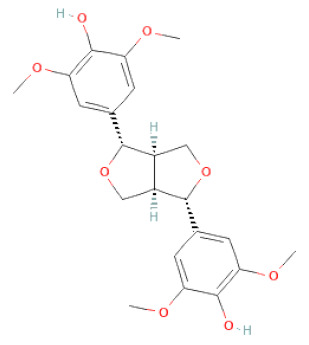	[30]
		Isolariciresinol	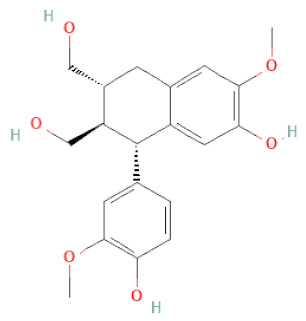	[30]
		Prestegane B	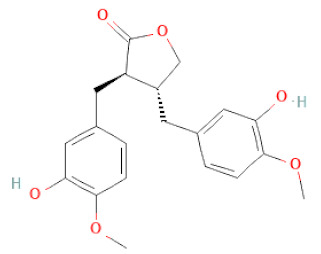	[33]
Non-flavonoids	Pterocarpans	(6aR,11aR)-maackiain	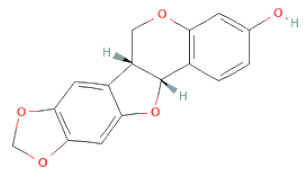	[30]
		(6aR,11aR)-medicarpin	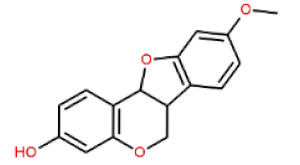	[30]
	Terpenoids	Lupeol	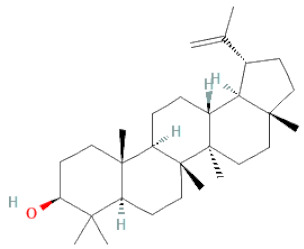	[33]
		Lupeone	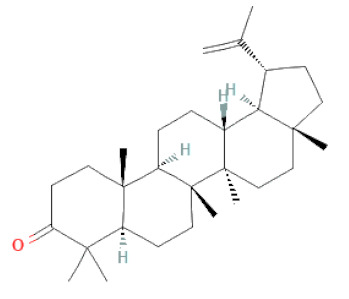	[33]
		Betulinic Acid	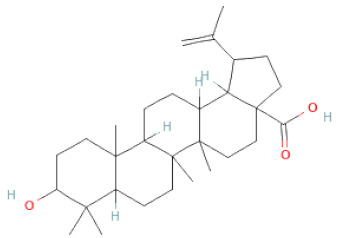	[19]
		Glycyrrhizin	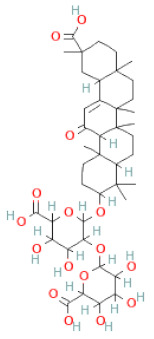	[27]
	Steroids	β-sitosterol	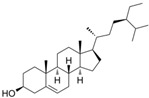	[20]
		β-sitosterone	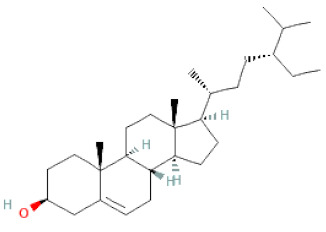	[19]
		Daucosterol	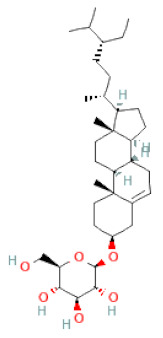	[30]
	Anthraquinones	Aloe-emodin	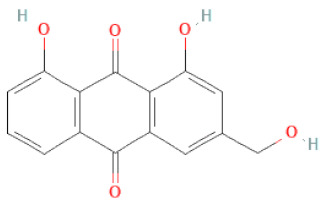	[30]
		Physcion (emodin-3-methyl ether)	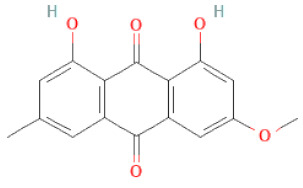	[33]
		Chrysophanol	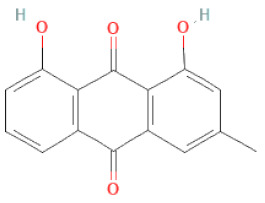	[33]
		Emodin	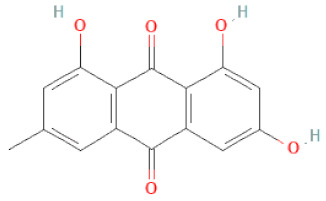	[20]
		Rhein	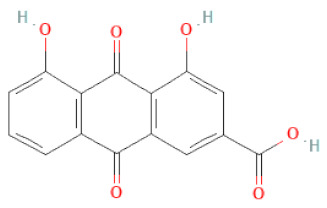	[19]
	Lactones	Angelicin	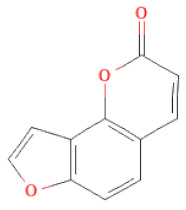	[30]
		n-butyl-O-β-D-fructopyranoside	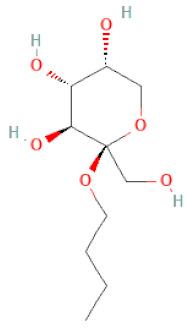	[30]
	Glycosides	2-methoxy-4-(2′-ethoxyl)-phenol-1-O-β-D-glucopyranosi de	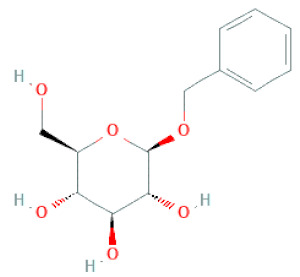	[33]
		5-O-(β-apiosyl-(1→2)-O-β-xylopyranosyl)gentisic acid	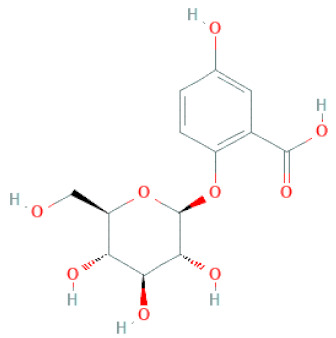	[33]
		15-O-(α-rhamnopyranosyl)-aloe-emodin	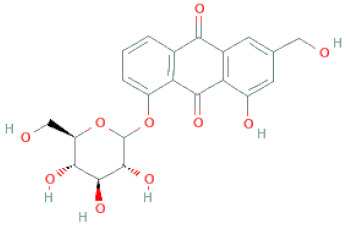	[20]
		1-O-(β-apiosyl-(1→6)-O-β-glucopyranosyl)-3-O-methyl phloroglucinol	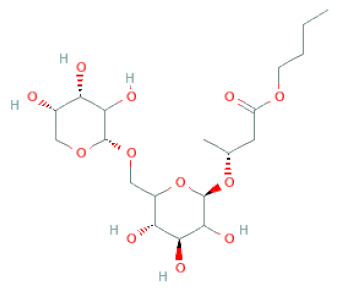	[19]
Other polyphenols	Furanocoumarins	5,7-Dihydroxycoumarin	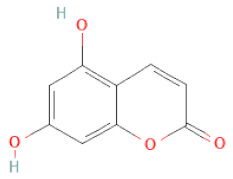	[30]
	glyoxylic acid	Allantoin	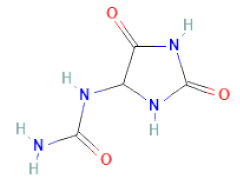	[30]
	Phenethyl alcohol	Benzene ethanol	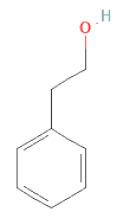	[33]
Phenolic acids	Hydroxybenzoic acids	3,4-dihydroxybenzoic acid	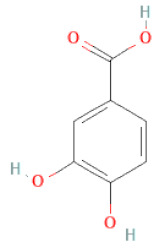	[34,36,37,38]
		3,4-dihydroxybenzaldehyde	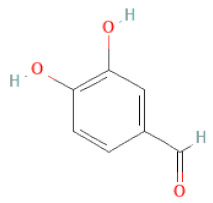	[34,36,37,38]
		β-glucogallin	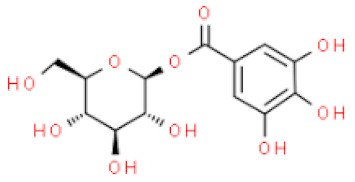	[34,36,37,38]
		*p*-hydroxy benzoic acid	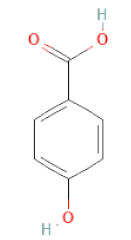	[27,30]
		Protocatechuic acid	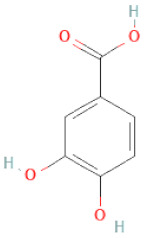	[30]
		Protocatechuic acid ethyl ester	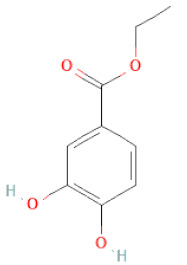	[33]
		Protocatechuic acid methyl ester	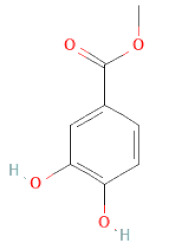	[30]
		Syringic acid	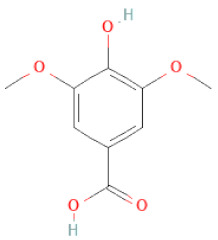	[20]
		Vanillic acid	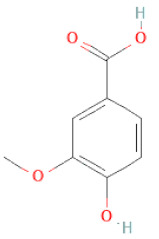	[20]
Saponins	Phytosterol	Daucosterol	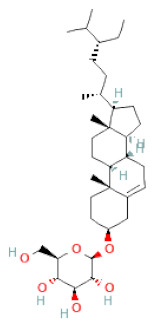	[20]

**Table 2:** In vitro anticancer activities of SSD.

Active Constituents of SSD	Dose	Positive Control	Mechanism of Action	Activity	References
(3S)-7-hydroxy-8,2′,4′-trimethoxyisoflavane, (3S)-7-hydroxy-8,2′-dimethoxy-4′,5′-methylenedioxyisoflavane, (S)-sativan and maackiain.	25.1–93.6 μM (IC50)	-	-	Anticancer activity	[40]
7,2′,4′-trihydroxy-8,3′-dimethoxyisoflavan	>40 μM (IC50)	Paclitaxel, cisplatin	-		[41]
7,4′-dihydroxy-8,2′,3′-trimethoxyisoflavan	>19.11 μM (IC50)	Paclitaxel, cisplatin	-		[41]
7-Hydroxyflavanone	5.21 μM	Epoximicin (IC50 = 65 nM)	20S proteasome inhibition		[69]
Aqueous extract	1000 μg/mL	-	-		[96]
	50–300 μg/mL	Docetaxel	G2/M checkpoint; Apoptosis induction; ROS induction		[94]
	50–300 μg/mL	-	BC cell apoptosis; G2/M phase arrest; ROS accumulation and inhibition of LDH-A		[118]
	0.07 μg/mL	-	-		[122]
	5–20 mg/plate	-	-		[126]
CHCl3-soluble and EtOAc-soluble fractions	100 µg/mL	Epoximicin (IC50 = 65 nM)	20S proteasome inhibition		[69]
EGC	60–100 μM	-	LDH-A, HIF-1α		[118]
Ethanol (60% (*v*/*v*) in water) extracts	10.89–52.58 μg/mL (IC50)	Docetaxel	ROS induced pyroptosis		[119]
Ethanol extracts	10–20 μg/mL	-	ROS induced apoptosis		[93]
Ethyl acetate fraction of methanol extract	7.5–15 mg/plate	-	-		[126]
Formononetin	>1000 μg/plate	-	-		[126]
Gallocatechin, catechin, epicatechin	-	-	-		[118]
Genistein	9.26 μM	Epoximicin (IC50 = 65 nM)	20S proteasome inhibition		[69]
Genistein and EGC	12.5–100 μg/mL	-	ROS induced apoptosis		[93]
Isoliquiritigenin	4.88 μM	Epoximicin (IC50 = 65 nM)	20S proteasome inhibition		[69]
Isoliquiritigenin analogues	0.71–7.9 μM (IC50)	Paclitaxel	-		[29]
Liquiritigenin, daidzein, medicarpin, and formononetin	>100 μM	Epoximicin (IC50 = 65 nM)	20S proteasome inhibition		[69]
Medicarpin, isoliquiritigenin, genistein and naringenin	100–500 μg/plate	-	Cell cycle inhibition; Antioxidant		[126]
Methanol extract	5–20 mg/plate	-	-		[126]
Sativan	10–100 μM	-	miR-200c/PD-L1 regulation; apoptosis induction		[46]
Sub-column extracts from ethanol (80% (*v*/*v*) in water) extracts	40–320 μg/mL	-	Reduced activity of ER and downregulation of PI3K/AKT and MAPK pathway		[27]
Kaempferol	28.8 ± 1.5 μM (with 1 nM dihydrotestosterone)	-	Inhibits the activation of androgen receptors		[127]
	58.3 ± 3.5 μM (with 1 nM dihydrotestosterone)	-	Apoptosis induction		
	38.35 ± 1.94 μM		Cell cycle inhibition; Antioxidant		[128]
	50 μM		Antioxidant, antimicrobial and cytotoxic activities		[129]
	78.4 μM (24 h), 38.1 μM (48 h)		Inhibition of DNA methylation		[130]
	54.7 μM		Promoted antioxidant enzymes; inhibited ROS generation and lipid peroxidation; inhibiting the function of phosphorylated AKT (p-AKT), CyclinD1, CDK4, Bid, Mcl-1, and Bcl-xL; promoted p-BRCA1, p-ATM, p53, p21, p38, Bax and Bid expression		[131]
Fisetin	34.1 ± 7.7 μM	Docetaxel	ROS induced apoptosis		[132]
	32.50 μM	-	Cell cycle arrest; antiproliferative effect		[133]
	20, 40 and 80 µM	-	Suppressed cell proliferation metastasis and invasiveness; induced the apoptosis		[134]
	10–100 μM	-	Suppressed cell proliferation by regulating PI3K/AKT/NF-κB		[135]
Myricetin	94.48 μM	-	Inhibiting PIM1 and disrupting the PIM1/CXCR4 interaction		[136]
	47.6 μM	-	Cell cycle inhibition; Antioxidant		[137]
	37.5–300 μM	-	Inhibition of cell proliferation		[138]
	10–100 μM	-	Promoted cell cycle arrest at G2/M, induced apoptosis, modulated Bcl-2 family proteins and activated caspase-3		[139]

*Abbreviation:* AKT—*protein* kinase B; AMPK—AMP-activated protein kinase; b.w.—body weight; EC50—Half maximal effective concentration; EGC—epigallocatechin; i.p.—intraperitoneal; IL-6—interleukin 6; MAPK—*mitogen-activated protein kinase;* PI3K—phosphoinositide 3-kinases; ROS—reactive oxygen species.

**Table 3:** In vivo anticancer activities of SSD.

Active Constituents of SSD	Model	Doses and Route of Administration	Positive Control	Mechanism of Action	Activity	References
Aqueous extract	BALB/c Nude mice	1 g/kg b.w., p.o.	Docetaxel	G2/M checkpoint; Apoptosis induction; ROS induction	Anti-cancer activity	[94]
	BALB/c Nude mice	1 g/kg b.w., p.o.	-	BC cell apoptosis; G2/M phase arrest; ROS accumulation and inhibition of LDH-A		[118]
EGC	BALB/c Nude mice	20, 40 mg/kg b.w, i.p.	-	LDH-A, HIF-1α		[118]
Ethanol (60% (*v*/*v*) in water) extracts	BALB/c Nude mice	0.8 g/kg b.w, p.o.	Docetaxel	ROS-induced pyroptosis		[119]
Sativan	BALB/c Nude mice	25, 50 mg/kg b.w, i.p. daily for 4 weeks	-	miR-200c/PD-L1 regulation; Apoptosis induction		[46]
Kaempferol	BALB/c nude mice	50, 100, 150 mg/kg b.w. p.o daily for 4 weeks	-	Suppression of tumor growth and metastasis; modulating DNA methylation by inhibiting DNMT3B		[130,140]
Fisetin	Athymic nude male mice; transgenic TRAMP mouse	20, 40 mg/kg; 3 times/week for 7 weeks	-	Tumor growth Inhibition by decreasing proliferation; inducing apoptosis; metastasis inhibition; synthesis and degradation inhibition of hyaluronan, an enzyme involved in cancer progression; overall survival increase		[141,142]
Myricetin	Wistar rats	25 mg/kg b.w. every 2 days for 40 days	-	Tumor growth inhibition; upregulation of p53 and downregulation of NF-κB pathway		[137]

*Abbreviation:* AKT—*protein* kinase B; AMPK—AMP-activated protein kinase; b.w.—body weight; EC50—Half maximal effective concentration; EGC—epigallocatechin; i.p.—intraperitoneal; IL-6—interleukin 6; MAPK—*mitogen-activated protein kinase;* p.o.—per oral; PI3K—phosphoinositide 3-kinases; ROS—reactive oxygen species.

## Data Availability

Not applicable.

## Supplementary Materials

The following supporting information can be downloaded at https://www.mdpi.com/article/10.3390/cells11182885/s1, Table S1: In vitro pharmacological properties of *Spatholobi Caulis* Dunn, Table S2. In vivo pharmacological properties of *Spatholobi Caulis* Dunn. References [178,179,180,181,182] are cited in the Supplementary Materials.
