# Supplementary material for: A Review of the Pharmacological Potential of Spatholobus suberectus Dunn on Cancer
Source: Cells. 2022 Sep 15;11(18):2885. doi: 10.3390/cells11182885 (PMC9497142; doi:10.3390/cells11182885)
Supplement: Supplementary file 1 [file cells-11-02885-s001.zip › cells-1925061-supplementary.pdf]

Supplementary Materials

A Review of the Pharmacological Potential of *Spatholobus suberectus* Dunn on Cancer

Feng Zhang[a,b#], Kumar Ganesan[a#], Qingqing Liu[a#], Jianping Chen[a,b*]

[a]School of Chinese Medicine, LKS Faculty of Medicine, The University of Hong Kong, Hong Kong
[b]Shenzhen Institute of Research and Innovation, The University of Hong Kong, Shenzhen, China

Table S1. *In vitro* pharmacological properties of *Spatholobi Caulis* Dunn

Active constituents of SSD	Dose	Positive control	Mechanism of action	Activity	References
Methanol extracts	200 μg/mL	-	HIV-1 PR inhibition	Antimicrobial activity	[1]
Aqueous extracts	14.8-60.8 μg/mL	-	-		[2]
	5, 10 μg/mL	-	-		[3]
Ethanol (0-75% (v/v) in water) extracts	0.45-1.13 μg/mL (EC50)	-	HCV replication suppression		[4]
ECG, EGCG	~68 μM (EC50)	-	HCV replication suppression		[4]
7-hydroxy-6-methoxy-flavanone	96,1 μM (IC50)	-	Sortase A (SrtA) activity inhibition		[5]
Formononetin	74.9 μM (IC50)	-	Sortase A (SrtA) activity inhibition		[5]

Ethanol (80% (v/v) in water) extracts	0.195-1.56 mg/mL (MIC)	Oxacillin (32 µg/mL) and ampicillin (4 µg/mL)	-		[6]
Ethanol (95% (v/v) in water) extracts	83.9 µg/mL (IC50)	-	Inhibitory effects on tyrosinase		[7]
	98.14 µM ascorbate equivalent /g	Ascorbic acid	DPPH scavenging activity		[8]
Methanol extracts	3.3 µg/mL (IC50)	Ascorbic acid and trolox	Hydrogen peroxide inhibition		[9]
Formononetin	10 µM	-	Inhibition of early-phase apoptosis		[10]
Aqueous extracts	1990 mM Trolox equivalent /g	Trolox	-		[11]
	184.4 µM ascorbate equivalent /g	Ascorbic acid	DPPH scavenging activity	Antioxidant activity	[8]
	-	Deferoxamine (10 mM)	ROS scavenging		[12]
	6.42 µg/mL, IC50	Ascorbic acid, acarbose	ROS scavenging; Down regulation of PI3K/AKT and AMPK Pathways		[13,14]
	30-100 µg/mL	EGCG	The inhibition of elastase enzyme activity		[15]

Ethanol extracts	25-100 µg/mL	Ascorbic acid	ROS scavenging		[16]
	2.81 µg/mL, IC50	Ascorbic acid, acarbose	ROS scavenging; Down regulation of PI3K/AKT and AMPK Pathways		[14,17]
	6-40 µg/ml	Amifostine (45 mg/kg)	Decreased levels of ROS and MDA: Increased levels of SOD, GPX, and G-CSFR		[18]
	30-100 µg/mL	EGCG	Decreased signaling of MMPs, TIMP-1, COL1A1, elastin, and HAS2; MAPK/AP-1; NF-κB and c-Jun		[15]
Ethanol (70% (v/v) in water) extracts	10-100 µg/mL	-	Increase Nrf2/ARE the stress-response system		[19]
Combined actions of epicatechin, syringic acid, and vanillic acid	2:1:1 (ratio of syringic acid, vanillic acid, and epicatechin)	-	The synergistic inhibition effect on elastase activity		[15]
Catechin	10 µM	-	-		[20]

Daidzein, formononetin, and procyanidin B2	-	-	-	Stimulation of hematopoiesis	[20]
Sub-fractions of ethanol extracts	31-631 μg/mL (IC50)	P. kadsura	Inhibition of cyclooxygenase-1/2, phospholipase A2, 5-lipoxygenase and 12-lipoxygenase	Anti-inflammatory activity	[21]
Aqueous extracts	60 μg/mL (IC50)	-	Inhibition of NO and TNF-α production		[8,22]
Total phenolics	1.33 μg/mL (IC50)	Sivelestat	-		[23]
Catechin, gallocatechin, liquiritigenin, , butin, 3',4',7-trihydroxyflavone, and plathymenin	-	-	5-Lipoxygenase inhibition		[24]
2,6-dimethoxy-1,4-benzoquinone, isoliquiritigenin	5.69-6.78 μM	l-N6-(1-iminoethyl)-lysine), indomethacin	Inhibition of NO; Pro-inflammatory cytokine inhibition		[25]
Spasuberol C, formononetin sodium, liquiritigenin, genistein, formononetin	16.34-28.33 μM	l-N6-(1-iminoethyl)-lysine), indomethacin	Inhibition of NO; Pro-inflammatory cytokine inhibition		[25]

Spasuberol A and B, daidzein, genistin, 4,7,2-trihydroxy-4-methoxy-isoflavanol, and homovanillyl-4-oxo-nonanoate	36.43-46.26 μM	l-N6-(1-iminoethyl)-lysine), indomethacin	Inhibition of NO; Pro-inflammatory cytokine inhibition		[25]
Spasuberoside A, spasuberoside B	> 100 μM	l-N6-(1-iminoethyl)-lysine), indomethacin	Inhibition of NO; Pro-inflammatory cytokine inhibition		[25]
8-O-methylretusin, biochanin A, formononetin, genistin, homovanillyl-4-oxo-nonanoate, odoratin, and Spasuberol A	21.1-51.1 μM	l-N6-(1-iminoethyl)-lysine), indomethacin	Inhibition of NO		[26]
(+)-epipinoresinol, (+)-pinoresinol, 3-methoxydaidzein, 4-hydroxy-3-methoxy cinnamic acid, methyl ester, biochanin A, butesuperin A, calycosin, daidzin, isolariciresinol, maackiain, ononin, and Salicylic acid	57.8-197.4 μM	l-N6-(1-iminoethyl)-lysine), indomethacin	Inhibition of NO		[26]
4,8-dimethoxy7-O-β-d-glucopyranosyl isoflavone, 6,9-dihydroxy megastigma-4,7-	> 200 μM	l-N6-(1-iminoethyl)-lysine), indomethacin	Inhibition of NO		[26]

dien-3-one, 8,9-dihydroxy megastigma-4,6-dien-3-one, nicotinic acid, phydroxybenzoic acid, and protocatechuate					
Procyanidin B4	22.5 nM (IC50)	Etoposide (IC50 = 78.4 μM)	-	Topoisomerase-II-inhibitory activity	[27]
(+)-catechin-(4→8)-(+)-catechin-(4→8)-(-)-epicatechin	21.9 nM (IC50)	Etoposide (IC50 = 78.4 μM)	-		[27]
Methanol extract	100-500 μg/mL	Ketoconazole (10 μM)	Cyt P450 inhibition	Cyt P450 3A4 enzyme inhibition *activity*	[28]
Aqueous extract	100-1000 μg/mL	Ketoconazole (10 μM)	Cyt P450 inhibition		[28]
Aqueous extracts	10-100 μg/mL	-	Suppression of c-Fos, NFATc1; The block of receptor activator of NF-κB ligand-induced early signaling pathways	Inhibition of osteoclast differentiation and bone resorption	[29]
Ethanol (70% (v/v) in water) extracts	12.5-200 μg/mL	Baicalein (10-20 μM)	-		[30]
Aqueous and ethanolic extracts	3, 10, 30 μg/mL	Acarbose (125-500 μg/mL)	Inhibition of *α*-glucosidase activity	Anti-diabetic activity	[14]
	10, 50, 100 μg/mL	Glucose and fructose (25 mM)	Inhibition of *AGEs formation*		[19]

Abbreviation: AGEs- advanced glycation end products; AKT- *protein* kinase B; AMPK- AMP-activated protein kinase; AP-1- activator protein 1; ARE- antioxidant responsive element; b.w. body weight; BPA- production of erythroid burst-promoting activity; c-Jun-transcription factor Jun; COL1A1- type I collagen; Cyt P450- cytochrome P450; DPPH- 2,2-diphenyl-1-picrylhydrazyl; EC50- Half maximal effective concentration; EGC- epigallocatechin; EGCG-epigallocatechin gallate; G-CSFR- granulocyte colony-stimulating factor receptor; GM-CSA- granulocyte-monocyte colony-stimulating activity; GM-CSF- granulocyte-macrophage colony-stimulating factor; GPX- glutathione peroxidase; HAMP- hepcidin Antimicrobial Peptide; HAS2- hyaluronan synthase 2; HCV- hepatitis C virus; HIV 1 PR- human immune deficiency virus-1 protease; i.p. intraperitoneal; IL-6 –interleukin 6; MAPK- *mitogen-activated protein kinase;* MDA- malondialdehyde; MIC- minimum inhibitory concentration; MK-CSA- megakaryocyte colony-stimulating activity; MMPs- matrix metalloproteinases; NFATc1- nuclear factor of activated T-cells; NF-κB - nuclear factor kappa B; NO- nitric oxide; Nrf2- nuclear factor erythroid 2-related factor 2; p.o. per oral; PI3K- phosphoinositide 3-kinases; ROS- reactive oxygen species; SOD- superoxide dismutase; TIMP-1- tissue inhibitor matrix metalloproteinase 1; TNF-α- tumor necrosis factor-alpha

Table S2. *In vivo* pharmacological properties of *Spatholobi Caulis* Dunn

Active constituents of SSD	Model	Doses and route of administration	Positive control	Mechanism of action	Activity	References
Aqueous extracts	BALB/c mice	50, 100 mg/kg b.w., p.o.	-	-	Antimicrobial activity	[3]
Aqueous extracts	Wistar albino rats	100, 200 mg/kg, p.o.	-	Decrease NO, MDA, TNF-α, caspase-3, and NF-κB; Increase GPX, SOD, and IL-10 expressions	Antioxidant activity	[31]
	C57BL/6J mice	200 mg/kg b.w. p.o.	Ascorbic acid, acarbose	ROS scavenging; Down regulation of PI3K/AKT and AMPK Pathways		[13,14]
Ethanol extracts	C57BL/6J mice	200 mg/kg b.w. p.o.	Ascorbic acid, acarbose	ROS scavenging; Down regulation of PI3K/AKT and AMPK Pathways		[14,17]
	Kun Ming mice	40 g/kg b.w, p.o.	Amifostine (45 mg/kg)	Decreased levels of ROS and MDA: Increased levels of SOD, GPX, and G-CSFR		[18]
Ethanol (70% (v/v) in water) extracts	db/db, C57BL/KsJ and db/m mice	200 mg/kg b.w., p.o.	-	Increase Nrf2/ARE the stress-response system		[19]
Catechin	Kun Ming mice	1 mg/day, i.p.	-	GM-CSF; IL-6	Stimulation of hematopoiesis	[32]
	NIH strain mice	0.01, 0.05 g/kg b.w, i.p.	-	GM-CSA, BPA and MK-CSA		[33]

Decoction of SSD	Kun Ming mice	-	-	-		[34]
Aqueous extracts	C57BL/6 female mice	108 g dried CS/kg b.w., i.p.	-	HAMP expression inhibition; Decrease in hepatic iron concentration; Increase in serum iron concentration		[35]
Aqueous extracts	Wistar albino rats	100, 200 mg/kg b.w., p.o.	Heparin	Decreased IL-Iβ and amylase and lipase	Anti-inflammatory activity	[36]
Liquiritigenin	BALB/c mice	1 mg/mL	-	Blocked T Cell Activation; Downregulation of PI3K/Akt pathways		[37]
Ethanol (95% (v/v) in water) extracts	ICR mice	100 and 200 mg/kg b.w., p.o.	Aspirin (1 mM), nitroprusside 30 (μg/ml)	Prevention of fibrinogen binding to GP IIb/IIIa receptor	Antiplatelet effects	[38]
Aqueous extracts	SD rats	10 mg/kg b.w., p.o.	Ketoconazole (0.25 and 1 μM)	Cyt P450 inhibition	Hepatoprotective activity	[39]
Aqueous and ethanolic extracts	C57BL/6J mice	100 and 200 mg/kg b.w., p.o	STZ-induced diabetic plus acarbose (200 mg /kg bw., p.o.)	Reduced blood glucose, oral glucose tolerance test, Phosphoenol pyruvate carboxykinase, and glucose-6-phosphatase, enhances glucose uptake and GLUT4 activation via PI3K/AKT and AMPK pathway	Anti-diabetic activity	[14]

C57BL/ KsJ db/db mice and db/m mice	50 mg/kg b.w., p.o	Metfor min (200 mg/kg, p.o.)	Reduced body weight, blood glucose, HbA1c, triglyceride, total cholesterol, low-density lipoprotein cholesterol, and free fatty acid		[19]

Abbreviation: AKT- *protein* kinase B; AMPK- AMP-activated protein kinase; AP-1- activator protein 1; ARE- antioxidant responsive element; b.w. body weight; BPA- production of erythroid burst-promoting activity; c-Jun-transcription factor Jun; COL1A1- type I collagen; Cyt P450- cytochrome P450; DPPH- 2,2-diphenyl-1-picrylhydrazyl; EC50- Half maximal effective concentration; EGC- epigallocatechin; EGCG-epigallocatechin gallate; G-CSFR- granulocyte colony-stimulating factor receptor; GM-CSA- granulocyte-monocyte colony-stimulating activity; GM-CSF- granulocyte-macrophage colony-stimulating factor; GPX- glutathione peroxidase; HAMP- hepcidin Antimicrobial Peptide; HAS2- hyaluronan synthase 2; HCV- hepatitis C virus; HIV 1 PR- human immune deficiency virus-1 protease; i.p. intraperitoneal; IL-6 –interleukin 6; MAPK- *mitogen-activated protein kinase;* MDA- malondialdehyde; MIC- minimum inhibitory concentration; MK-CSA- megakaryocyte colony-stimulating activity; MMPs- matrix metalloproteinases; NFATc1- nuclear factor of activated T-cells; NF-κB - nuclear factor kappa B; NO- nitric oxide; Nrf2- nuclear factor erythroid 2-related factor 2; p.o. per oral; PI3K- phosphoinositide 3-kinases; ROS- reactive oxygen species; SOD- superoxide dismutase; TIMP-1- tissue inhibitor matrix metalloproteinase 1; TNF-α- tumor necrosis factor-alpha

References

1. Lam, T.L.; Lam, M.L.; Au, T.K.; Ip, D.T.; Ng, T.B.; Fong, W.P.; Wan, D.C. A comparison of human immunodeficiency virus type-1 protease inhibition activities by the aqueous and methanol extracts of Chinese medicinal herbs. *Life Sci.* **2000**, *67*, 2889-2896, doi:10.1016/s0024-3205(00)00864-x.
2. Guo, J.P.; Pang, J.; Wang, X.W.; Shen, Z.Q.; Jin, M.; Li, J.W. In vitro screening of traditionally used medicinal plants in China against enteroviruses. *World J. Gastroenterol.* **2006**, *12*, 4078-4081, doi:10.3748/wjg.v12.i25.4078.
3. Pang, J.; Guo, J.P.; Jin, M.; Chen, Z.Q.; Wang, X.W.; Li, J.W. Antiviral effects of aqueous extract from Spatholobus suberectus Dunn. against coxsackievirus B3 in mice. *Chin J Integr Med* **2011**, *17*, 764-769, doi:10.1007/s11655-011-0642-1.
4. Chen, S.R.; Wang, A.Q.; Lin, L.G.; Qiu, H.C.; Wang, Y.T.; Wang, Y. In Vitro Study on Anti-Hepatitis C Virus Activity of Spatholobus suberectus Dunn. *Molecules* **2016**, *21*, doi:10.3390/molecules21101367.
5. Park, W.; Ahn, C.H.; Cho, H.; Kim, C.K.; Shin, J.; Oh, K.B. Inhibitory Effects of Flavonoids from Spatholobus suberectus on Sortase A and Sortase A-Mediated Aggregation of Streptococcus mutans. *Journal of Microbiolog and Biotechnology* **2017**, *27*, 1457-1460, doi:10.4014/jmb.1704.04001.
6. Kim, G.; Gan, R.Y.; Zhang, D.; Farha, A.K.; Habimana, O.; Mavumengwana, V.; Li, H.B.; Wang, X.H.; Corke, H. Large-Scale Screening of 239 Traditional Chinese Medicinal Plant Extracts for Their Antibacterial Activities against Multidrug-Resistant Staphylococcus aureus and Cytotoxic Activities. *Pathogens* **2020**, *9*, doi:10.3390/pathogens9030185.
7. Wang, K.H.; Lin, R.D.; Hsu, F.L.; Huang, Y.H.; Chang, H.C.; Huang, C.Y.; Lee, M.H. Cosmetic applications of selected traditional Chinese herbal medicines. *J. Ethnopharmacol.* **2006**, *106*, 353-359, doi:10.1016/j.jep.2006.01.010.
8. Ravipati, A.S.; Zhang, L.; Koyyalamudi, S.R.; Jeong, S.C.; Reddy, N.; Bartlett, J.; Smith, P.T.; Shanmugam, K.; Munch, G.; Wu, M.J.; et al. Antioxidant and anti-inflammatory activities of selected Chinese medicinal plants and their relation with antioxidant content. *BMC Complement. Altern. Med.* **2012**, *12*, 173, doi:10.1186/1472-6882-12-173.
9. Chang, C.L.; Lin, C.S.; Lai, G.H. Phytochemical characteristics, free radical scavenging activities, and neuroprotection of five medicinal plant extracts. *Evid. Based Complement. Alternat. Med.* **2012**, *2012*, 984295, doi:10.1155/2012/984295.
10. Tian, Z.; Liu, S.B.; Wang, Y.C.; Li, X.Q.; Zheng, L.H.; Zhao, M.G. Neuroprotective effects of formononetin against NMDA-induced apoptosis in cortical neurons. *Phytother. Res.* **2013**, *27*, 1770-1775, doi:10.1002/ptr.4928.
11. Liao, H.; Banbury, L.K.; Leach, D.N. Antioxidant activity of 45 Chinese herbs and the relationship with their TCM characteristics. *Evid. Based Complement. Alternat. Med.* **2008**, *5*, 429-434, doi:10.1093/ecam/nem054.
12. Toyama, T.; Wada-Takahashi, S.; Takamichi, M.; Watanabe, K.; Yoshida, A.; Yoshino, F.; Miyamoto, C.; Maehata, Y.; Sugiyama, S.; Takahashi, S.S.; et al. Reactive oxygen species scavenging activity of Jixueteng evaluated by electron spin resonance (ESR) and photon emission. *Nat Prod Commun* **2014**, *9*, 1755-1759.
13. Fu, Y.F.; Jiang, L.H.; Zhao, W.D.; Xi-Nan, M.; Huang, S.Q.; Yang, J.; Hu, T.J.; Chen, H.L. Immunomodulatory and antioxidant effects of total flavonoids of Spatholobus suberectus Dunn on PCV2 infected mice. *Sci Rep* **2017**, *7*, 8676, doi:10.1038/s41598-017-09340-9.
14. Zhao, P.; Alam, M.B.; Lee, S.H.; Kim, Y.J.; Lee, S.; An, H.; Choi, H.J.; Son, H.U.; Park, C.H.; Kim, H.H.; et al. Spatholobus suberectus Exhibits Antidiabetic Activity In Vitro and In

Vivo through Activation of AKT-AMPK Pathway. *Evid Based Complement Alternat Med* **2017**, *2017*, 6091923, doi:10.1155/2017/6091923.
15. Kwon, K.R.; Alam, M.B.; Park, J.H.; Kim, T.H.; Lee, S.H. Attenuation of UVB-Induced Photo-Aging by Polyphenolic-Rich Spatholobus Suberectus Stem Extract Via Modulation of MAPK/AP-1/MMPs Signaling in Human Keratinocytes. *Nutrients* **2019**, *11*, doi:10.3390/nu11061341.
16. Tan, X.; Dong, X.Z.; Guo, D.H.; Wang, S.; Li, M.H.; Zhao, R.Q.; Liu, P. [Anti-radiation effect and mechanism studies of ethanol extracts from Spatholobus suberectus and its active component catechin]. *Zhongguo Zhong Yao Za Zhi* **2016**, *41*, 1718-1724, doi:10.4268/cjcmm20160924.
17. Chen, H.L.; Yang, J.; Fu, Y.F.; Meng, X.N.; Zhao, W.D.; Hu, T.J. Effect of total flavonoids of Spatholobus suberectus Dunn on PCV2 induced oxidative stress in RAW264.7 cells. *BMC Complement Altern Med* **2017**, *17*, 244, doi:10.1186/s12906-017-1764-6.
18. Dong, X.Z.; Wang, Y.N.; Tan, X.; Liu, P.; Guo, D.H.; Yan, C. Protective Effect of JXT Ethanol Extract on Radiation-Induced Hematopoietic Alteration and Oxidative Stress in the Liver. *Oxid. Med. Cell. Longev.* **2018**, *2018*, 9017835, doi:10.1155/2018/9017835.
19. Do, M.H.; Hur, J.; Choi, J.; Kim, Y.; Park, H.Y.; Ha, S.K. Spatholobus suberectus Ameliorates Diabetes-Induced Renal Damage by Suppressing Advanced Glycation End Products in db/db Mice. *Int J Mol Sci* **2018**, *19*, doi:10.3390/ijms19092774.
20. Chang, J.; Sun, W.; Zeng, J.; Xue, Y.; Zhang, Y.; Pan, X.; Zhou, Y.; Lai, M.; Bian, G.; Zhou, Q.; et al. Establishment of an in vitro system based on AGM-S3 co-culture for screening traditional herbal medicines that stimulate hematopoiesis. *J. Ethnopharmacol.* **2019**, *240*, 111938, doi:10.1016/j.jep.2019.111938.
21. Li, R.W.; David Lin, G.; Myers, S.P.; Leach, D.N. Anti-inflammatory activity of Chinese medicinal vine plants. *J. Ethnopharmacol.* **2003**, *85*, 61-67, doi:10.1016/s0378-8741(02)00339-2.
22. Cai, Y.; Wang, S.; Guo, W.; Xie, Z.; Zheng, Y.; Cao, Z.; Zhou, Y. Transcriptome analysis provides insights into the immune responsive pathways and genes in the head kidney of tiger grouper (Epinephelus fuscoguttatus) fed with Spatholobus suberectus, Phellodendron amurense, or Eclipta prostrata. *Fish Shellfish Immunol.* **2018**, *73*, 100-111, doi:10.1016/j.fsi.2017.12.004.
23. Huang, Y.; Chen, L.; Feng, L.; Guo, F.; Li, Y. Characterization of total phenolic constituents from the stems of Spatholobus suberectus using LC-DAD-MS(n) and their inhibitory effect on human neutrophil elastase activity. *Molecules* **2013**, *18*, 7549-7556, doi:10.3390/molecules18077549.
24. Jiang, S.; Huang, K.; Liu, W.; Fu, F.; Xu, J. Combined Autodock and comparative molecular field analysis study on predicting 5-lipoxygenase inhibitory activity of flavonoids isolated from Spatholobus suberectus Dunn. *Zeitschrift fur Naturforschung. C, Journal of biosciences* **2015**, *70*, 103-113, doi:10.1515/znc-2014-4110.
25. Liu, X.Y.; Zhang, Y.B.; Yang, X.W.; Yang, Y.F.; Xu, W.; Zhao, W.; Peng, K.F.; Gong, Y.; Liu, N.F.; Zhang, P. Anti-Inflammatory Activity of Some Characteristic Constituents from the Vine Stems of Spatholobus suberectus. *Molecules* **2019**, *24*, doi:10.3390/molecules24203750.
26. Liu, X.Y.; Zhang, Y.B.; Yang, X.W.; Xu, W.; Liu, L.; Zhang, P.; Gong, Y.; Liu, N.F.; Peng, K.F. Simultaneous determination of twenty-five compounds with anti-inflammatory activity in Spatholobi Caulis by using an optimized UFLC-MS/MS method: An application to pharmacokinetic study. *Journal of Pharmaceutical and Biomedical Analysis* **2021**, *204*, 114267, doi:10.1016/j.jpba.2021.114267.

27. Han, A.R.; Park, H.J.; Chen, D.; Jang, D.S.; Kim, H.J.; Lee, S.K.; Seo, E.K. Topoisomerase-II-inhibitory principles from the stems of Spatholobus suberectus. *Chem. Biodivers.* **2007**, *4*, 1487-1491, doi:10.1002/cbdv.200790127.
28. Ashour, M.L.; Youssef, F.S.; Gad, H.A.; Wink, M. Inhibition of Cytochrome P450 (CYP3A4) Activity by Extracts from 57 Plants Used in Traditional Chinese Medicine (TCM). *Pharmacogn. Mag.* **2017**, *13*, 300-308, doi:10.4103/0973-1296.204561.
29. Ha, H.; Shim, K.S.; An, H.; Kim, T.; Ma, J.Y. Water extract of Spatholobus suberectus inhibits osteoclast differentiation and bone resorption. *BMC Complement. Altern. Med.* **2013**, *13*, 112, doi:10.1186/1472-6882-13-112.
30. Im, N.K.; Lee, S.G.; Lee, D.S.; Park, P.H.; Lee, I.S.; Jeong, G.S. Spatholobus suberectus inhibits osteoclastogenesis and stimulates chondrogenesis. *Am. J. Chin. Med.* **2014**, *42*, 1123-1138, doi:10.1142/S0192415X14500700.
31. Zhang, R.; Liu, C.; Liu, X.; Guo, Y. Protective effect of Spatholobus suberectus on brain tissues in cerebral ischemia. *American Journal of Translational Research* **2016**, *8*, 3963-3969.
32. Liu, P.; Wang, D.X.; Chen, R.Y.; Chen, M.L.; Yin, J.F.; Chen, G.Y. [Effect of catechin on bone marrow cell cycle and gene expression of hematopoietic growth factors]. *Yao Xue Xue Bao* **2004**, *39*, 424-428.
33. Wang, D.X.; Liu, P.; Chen, Y.H.; Chen, R.Y.; Guo, D.H.; Ren, H.Y.; Chen, M.L. Stimulating effect of catechin, an active component of Spatholobus suberectus Dunn, on bioactivity of hematopoietic growth factor. *Chin. Med. J. (Engl.)* **2008**, *121*, 752-755.
34. Chen, D.H.; Luo, X.; Yu, M.Y.; Zhao, Y.Q.; Cheng, Y.F.; Yang, Z.R. [Effect of Spatholobus suberectus on the bone marrow cells and related cytokines of mice]. *Zhongguo Zhong Yao Za Zhi* **2004**, *29*, 352-355.
35. Guan, Y.; An, P.; Zhang, Z.; Zhang, F.; Yu, Y.; Wu, Q.; Shi, Y.; Guo, X.; Tao, Y.; Wang, F. Screening identifies the Chinese medicinal plant Caulis Spatholobi as an effective HAMP expression inhibitor. *J. Nutr.* **2013**, *143*, 1061-1066, doi:10.3945/jn.113.174201.
36. Shao, Z. Spatholobus Suberectus Stem Extract Improves the Protective Effect of Heparin on Cerulein-Induced Pancreatitis. *Afr. J. Tradit. Complement. Altern. Med.* **2017**, *14*, 187-193, doi:10.21010/ajtcam.v14i3.20.
37. Lee, H.S.; Kim, E.N.; Jeong, G.S. Oral Administration of Liquiritigenin Confers Protection from Atopic Dermatitis through the Inhibition of T Cell Activation. *Biomolecules* **2020**, *10*, doi:10.3390/biom10050786.
38. Lee, B.J.; Jo, I.Y.; Bu, Y.; Park, J.W.; Maeng, S.; Kang, H.; Jang, W.; Hwang, D.S.; Lee, W.; Min, K.; et al. Antiplatelet effects of Spatholobus suberectus via inhibition of the glycoprotein IIb/IIIa receptor. *J. Ethnopharmacol.* **2011**, *134*, 460-467, doi:10.1016/j.jep.2010.12.039.
39. Pao, L.H.; Hu, O.Y.; Fan, H.Y.; Lin, C.C.; Liu, L.C.; Huang, P.W. Herb-drug interaction of 50 Chinese herbal medicines on CYP3A4 activity in vitro and in vivo. *Am. J. Chin. Med.* **2012**, *40*, 57-73, doi:10.1142/S0192415X1250005X.
